# Blm10-Based Compounds Add to the Knowledge of How Allosteric Modulators Influence Human 20S Proteasome

**DOI:** 10.1021/acschembio.4c00341

**Published:** 2025-02-05

**Authors:** Julia Witkowska, Małgorzata Giżyńska, Przemysław Karpowicz, Daria Sowik, Karolina Trepczyk, Fabian Hennenberg, Ashwin Chari, Artur Giełdoń, Karolina Pierzynowska, Lidia Gaffke, Grzegorz Węgrzyn, Elżbieta Jankowska

**Affiliations:** 1 Department of Biomedical Chemistry, Faculty of Chemistry, University of Gdańsk, Gdańsk 80-308, Poland; 2 Department of Structural Dynamics, Max-Planck-Institute for Biophysical Chemistry, Goettingen 37077, Germany; 3 Research Group for Structural Biochemistry and Mechanisms, Max-Planck-Institute for Biophysical Chemistry, Goettingen 37077, Germany; 4 Department of Theoretical Chemistry, Faculty of Chemistry, University of Gdańsk, Gdańsk 80-308, Poland; 5 Department of Molecular Biology, Faculty of Biology, University of Gdańsk, Gdańsk 80-308, Poland

## Abstract

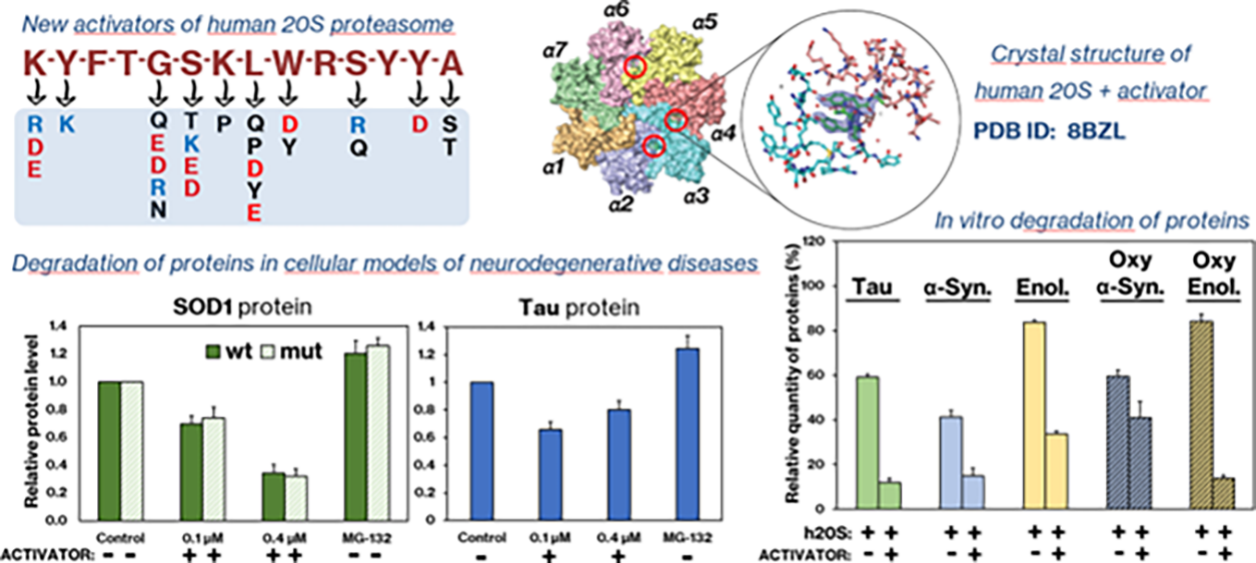

Proteasomes catalyze protein degradation in cells and play an integral role in cellular homeostasis. Its activity decreases with age alongside the load of defective proteins, resulting from mutations or oxidative stress-induced damage. Such proteins are prone to aggregation and, if not efficiently degraded, can form toxic oligomers and amyloid plaques. Developing an effective way to activate the proteasome could prevent such pathologies. Designing activators is not easy because they do not bind in the active site, which is well-defined and highly conserved, but away from it. The structures of proteasome complexes with natural activators can help here, but these are large proteins, some even multimeric, whose activity is difficult to replace with a small-molecule compound. Nevertheless, the use of fragments of such proteins makes it possible to accumulate knowledge about the relevance of various structural elements for efficient and selective activation. Here, we presented peptidic activators of the 20S proteasome, which were designed based on both the *C*-terminal sequence of the yeast proteasome activator, Blm10 protein, and the interactions predicted by molecular modeling. These Blm analogs were able to stimulate human 20S proteasome to more efficiently degrade both small fluorogenic substrates and proteins. The best activators also demonstrated their efficacy in cell lysates. X-ray crystallography indicated that an effective modulator can bind to several sites on the surface of the proteasome without causing permanent structural changes in its immediate vicinity but affecting the active sites.

## Introduction

From yeast to humans, proteasomes are present in all cells, where they play a central role in the degradation of the vast majority of intracellular proteins. Unfortunately, the proteasome activity decreases with age, which has serious consequences: cataract formation in the eye lens,^1^ skin aging,^2^ reduced tolerance of the aged heart to ischemia/reperfusion,^3^ and development of neurodegenerative diseases, such as the most abundant Alzheimer’s (AD) and Parkinson’s diseases (PD), Huntington’s disease, and amyotrophic lateral sclerosis (ALS).^4−6^ Due to aging populations, especially in developed countries, the number of incidents of these diseases is increasing dramatically. It worsens the quality of life of both the elderly and their caregivers and places a significant strain on medical services and national budgets. Therefore, the discovery of compounds that allow for efficient stimulation of proteasome activity and enable intervention in these pathologies is of great importance.^7^

The eukaryotic 20S proteasome (core particle, CP) is a barrel-shaped structure created by four stacked heptameric rings.^8−10^ The two outer rings comprise seven α-subunits (named α1-α7), while the two inner rings are composed of seven β-subunits (β1-β7). Each β-ring holds the catalytic sites with three different specificities: caspase-like (C-L), trypsin-like (T-L), and chymotrypsin-like (ChT-L) activities are displayed by the β1, β2, and β5 subunits, respectively. In the latent 20S proteasome, the channel leading to the active sites is closed by the *N*-terminal tails of the α-subunits, which point inward to the center of the ring, blocking access of substrates to the proteolytic chamber.^11^ Attachment of activating proteins: the dome-shaped heptameric 11S (PA28) activator complex,^12^ the homomeric activator PA200,^13^ or the ATP-dependent multimeric 19S regulatory cap^14^ is needed to open the gate. However, the role of activators is probably not limited to the gate regulation, but through their binding allosteric signals are transmitted, propagating to and stimulating the proteolytic sites.^15,16^

The 20S proteasome is capable of proteolysis of only fully or partially unfolded proteins, so its substrates, in addition to intrinsically disordered proteins, are proteins that have lost their native conformation due to mutation or oxidation. Therefore, this proteasome is responsible for so-called housekeeping chores, removing from cells abnormal proteins that otherwise could oligomerize and form pathological aggregates. The role of 26S proteasome is to take care of homeostasis by removing from cells proteins that have fulfilled their role and are no longer needed. These proteins when targeted for degradation are labeled by polyubiquitin chains, recognized by the 19S regulator attached to the catalytic core.

In contrast to proteasome inhibition, its activation by small molecules is a much less studied topic. Relatively few allosteric stimulators of the 20S proteasome have been described so far.

These are pyrazolones,^17^ chlorpromazine,^18^ TCH-165,^19^ AM-404,^20^ dihydroquinazolines,^21^ and fluspirilene analogs.^22^ An interesting trend in the search for effective activators is peptides and peptidomimetics derived from the proteins interacting with the proteasome’s α face. Activators of this type include the reported by us Tat-Den and Tat-8,9TOD modulators,^23^ based on a fragment of the HIV-1 Tat protein that competes with the 11S regulator for binding to the proteasome.^24^ To the same group belong short *C*-terminal fragments of the Rpt subunits of the 19S regulator and its *Archaea* ortholog PAN.^25,26^ Also, discovered by us, short analogs of the antibacterial peptide PR39 turned out to be quite efficient activators of h20S^27^.

A new leading structure can be Blm-pep.^28^ This peptide was designed based on the *C*-terminal sequence of the Blm10 protein, which is the yeast counterpart of the human PA200 activator. Blm10 and Blm-pep shared the HbYX motif (Hb-hydrophobic, Y-tyrosine, and X-any amino acid at the very *C*-terminus), which was demonstrated as responsible for anchoring both activators in the pocket between the α5 and α6 subunits of the proteasome.^28,29^ Research conducted so far established that the HbYX-dependent activators, such as 19S, PA200, and Blm10, use this motif not only to bind but also to induce gate-opening. Much attention has been paid to the mechanism of this opening, but the studies were frequently carried out in the simplified model of homo-oligomeric archaeal proteasomes.^26,30^ In addition, in many studies concerning the gate-opening mechanism, the HbYX was a part of natural or engineered multivalent proteinaceous activators.^31,32^ As Opoku-Nsiah et al. pointed out, one cannot be sure that proteasome does not have different requirements for monovalent activators.^32^ Furthermore, it is not known whether the mechanism of recognition of the HbYX motif in the archaeal system is strictly conserved in the human 20S, which contains seven distinct α pockets instead of being a homo-oligomer. Inspired by this, we decided to create a series of Blm-pep analogs that would allow us to better understand the structure–activity relationship of monovalent modulators of the human 20S proteasome.

We have proved previously by X-ray crystallography that Blm-pep binds to the yeast 20S (y20S) in a similar manner as Blm10.^28^ Despite binding, the peptidic modulator did not activate the enzyme. Dange et al. reported a similar failure in activating the y20S ChT-L peptidase for the 8-residue *C*-terminal fragment of Blm10.^33^ Interestingly, Blm-pep turned out to be an efficient activator of human 20S proteasome.^28^ This observation has motivated us to perform studies that could provide insight into the factors responsible for the activation of human enzyme. In the crystal structure of the y20S-Blm-pep complex, only five *C*-terminal amino acid residues of Blm-pep were visible, which suggested that only this fragment binds tightly and permanently to the proteasome. However, the biochemical tests demonstrated that the *N-*terminal part enhances activation of the core particle.^28^ Here, we present the results of our further investigation of the relationship between the modulator’s sequence and its capacity to stimulate the enzyme.

The activation potential of the compounds was probed using classic fluorogenic substrates. Besides, an elongated FRET-type substrate, DabEDS, and protein models were included in the tests of the modulators’ stimulating capacity. The best compounds were also tested in cell lysate. The site of binding of the best modulator was determined by X-ray crystallography.

## Results

### Design of Blm-pep Analogs

The crystal structure of the yeast 20S in complex with Blm-pep revealed its binding in the α5-α6 pocket.^28^ Anticipating the similar binding mode, we modeled Blm-pep in the same α5-α6 pocket of the human 20S proteasome (here and hereafter, α-subunits in h20S are labeled to match the numbering used in y20S) and designed a set of analogs, some of which were able to stimulate h20S up to five to six times.^34^ However, this small group of modulators did not allow for a more detailed analysis of the proteasome’s requirements for its allosteric activators. Besides, these modulators did not make use of the potential of the remaining binding pockets. Therefore, we utilized molecular modeling and “immersed” Blm-pep consecutively to each of the seven α pockets of h20S and designed new analogs by modifying the sequence of Blm-pep so that it can form contacts also within pockets other than the canonical α5−α6.

The parent peptide was able to anchor in almost all of the pockets of h20S thanks to the salt bridge formed between the C-terminal carboxyl group and a lysine residue (equivalent to Lys66 in archeal proteasome) (Figure S1). The only exceptions were the α7-α1 pocket, where no such residue is present, and the α6-α7 pocket, where the canonical Lys is substituted by Arg. Since there is not much space at the bottom of the pockets, the last residue was small, such as Ala, but also Ser and Thr, whose hydroxyl groups could potentially form a hydrogen bond and increase the stability of the ligand in the binding pocket. The residues at positions 13 and 12 were elements of the HbYX motif and therefore were not modified, except in one case, in which Tyr13Asp substitution was introduced to test whether the basic residues Arg20 and Lys28, present in the α5-α6 and α3-α4 pockets, respectively, could provide contacts that enhance the modulator’s activity. In some modulators, Ser11 was replaced with Arg because in most pockets, except for α5-α6 and α6-α7, a long and basic residue located at position 11 could find a salt bridge-forming partner. As the experimental structure showed,^28^ the side chain of Arg10 in the α5-α6 pocket of y20S faced the same direction as the side chain of Tyr12. Moreover, in our modeling, these residues were oriented in the same way in almost all of the pockets of the human enzyme, except for the α6-α7 and α7-α1, which at the same time do not contain the Lys residue to anchor the modulator (Figure S1). We noticed that at the upper part of most binding pockets, there were acidic residues, with which Arg can form a salt bridge. This interaction may act as a kind of a “pushing stick” against the Tyr12, placing it in the correct location at the bottom of the binding pocket. Since the proper position of residues from the HbYX motif was crucial to the mechanism of allosteric activation, the residue at position 10 was not exchanged. Close to the entrance of most pockets, there were patches of positive charge. Therefore, we inserted glutamic or aspartic acid in positions 8 and 9. In the pockets α5-α6, α6-α7, and α7-α1, a Tyr residue in position 9 would also find binding partners, so it was built into the sequence of some modulators. Modifications introduced to the Blm-pep sequence comprised also a turn-inducing Pro residue at position 7 or 8. The purpose of this modification was to force the modulator’s backbone to align along the α surface.

Although the design of modulators was guided by the crystal structures of 20S CP with Blm-pep and Blm10, residues providing optimal contacts between the enzyme and the modulator’s *N*-terminal segment could be directly derived from none of them. This was because the polypeptide chain extending beyond the five *C*-terminal residues was either not visible in the structure (20S-Blm-pep^28^) or moved away from the enzyme’s surface (20S-Blm10^29^). At the same time, we knew that the acetylated 5aa fragment of Blm-pep hardly activated h20S, but with the elongation of the *N*-terminal segment, this efficiency increased.^28^ To design the optimal sequence of the 1–6 region, we picked two directions in which the N-terminus of the modulator could potentially align (Figure S2A). The first one was a logical consequence of the mechanism of action of allosteric modulators, the binding of which opens the gate leading to the catalytic channel. As a likely binding place of the modulator’s N-terminal region, we chose the grooves that led from each of the binding pockets toward the gate (Figure S2B). The second direction was inspired by the postulated site of interaction of protein substrates with the 20S proteasome. Unstructured proteins were thought to interact with the projecting outward loop terminating the last helix of the α subunits. It has been reported, for instance, that retinoblastoma (Rb), p21, and Cdc25C proteins interact with the C-terminus of the α7 subunit, facilitating their own degradation.^35^ Therefore, the second potential direction of modulator binding was the groove between the outer surfaces of the α subunits (Figure S2C). To design modulators, we introduced to their sequences modifications that could provide favorable interactions in at least one of the potential binding directions.

Guided by the results of molecular modeling, we synthesized 25 Blm-pep-derived compounds. In one set of analogs, we modified solely the *C*-terminal fragment of Blm-pep, introducing different residues into positions 8–14; in the second one, we introduced substitutions only in positions 1–7 (Table 1, peptides **10**–**19** and **1**–**9**, respectively). In the third series, we combined modification in the *N*- and *C*-terminal segments (peptides **20**–**25**).

**Table 1:**
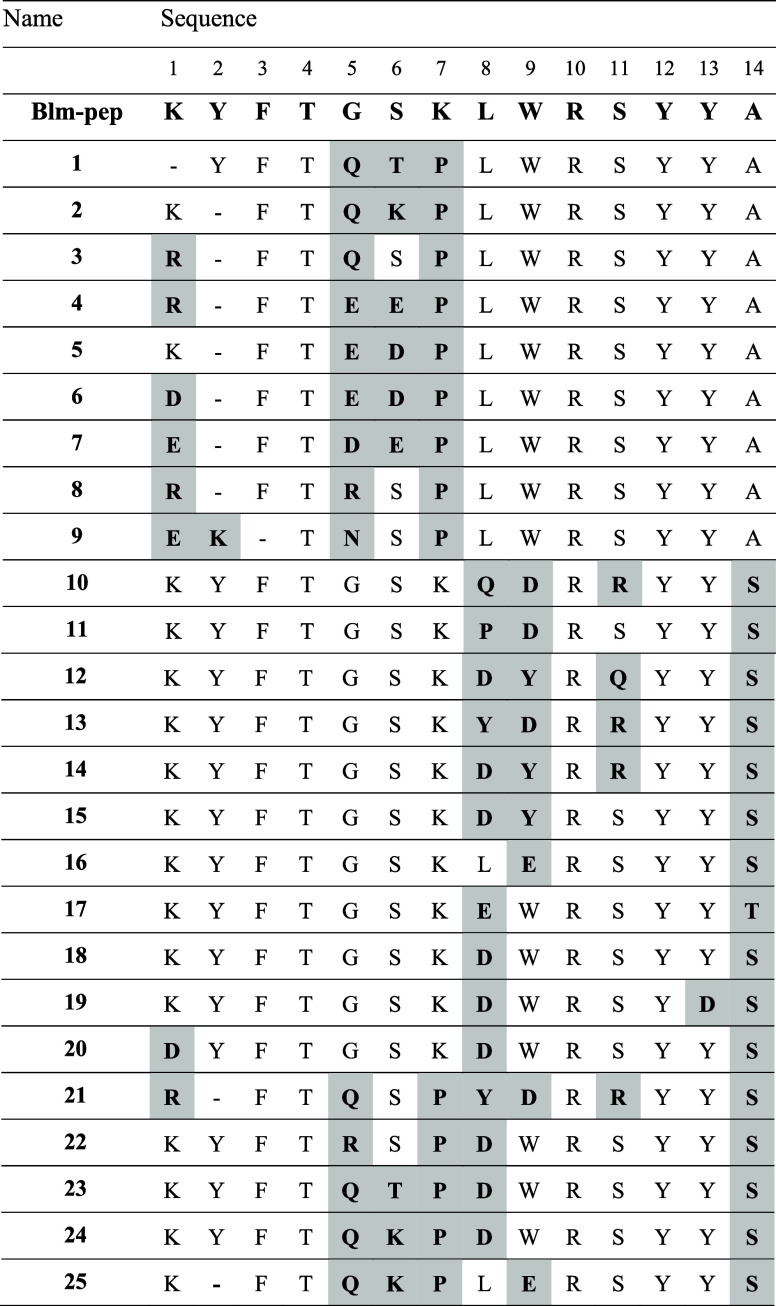
Sequences of Blm-pep Analogs, Which Were Inspired by the Interactions That the Modulator Could Create within and in the Immediate Vicinity of the Binding Pockets^a^

a Modified residues are shown in bold on a shaded background

### Activating Capacity of Blm-pep Analogs

**Figure 1:**
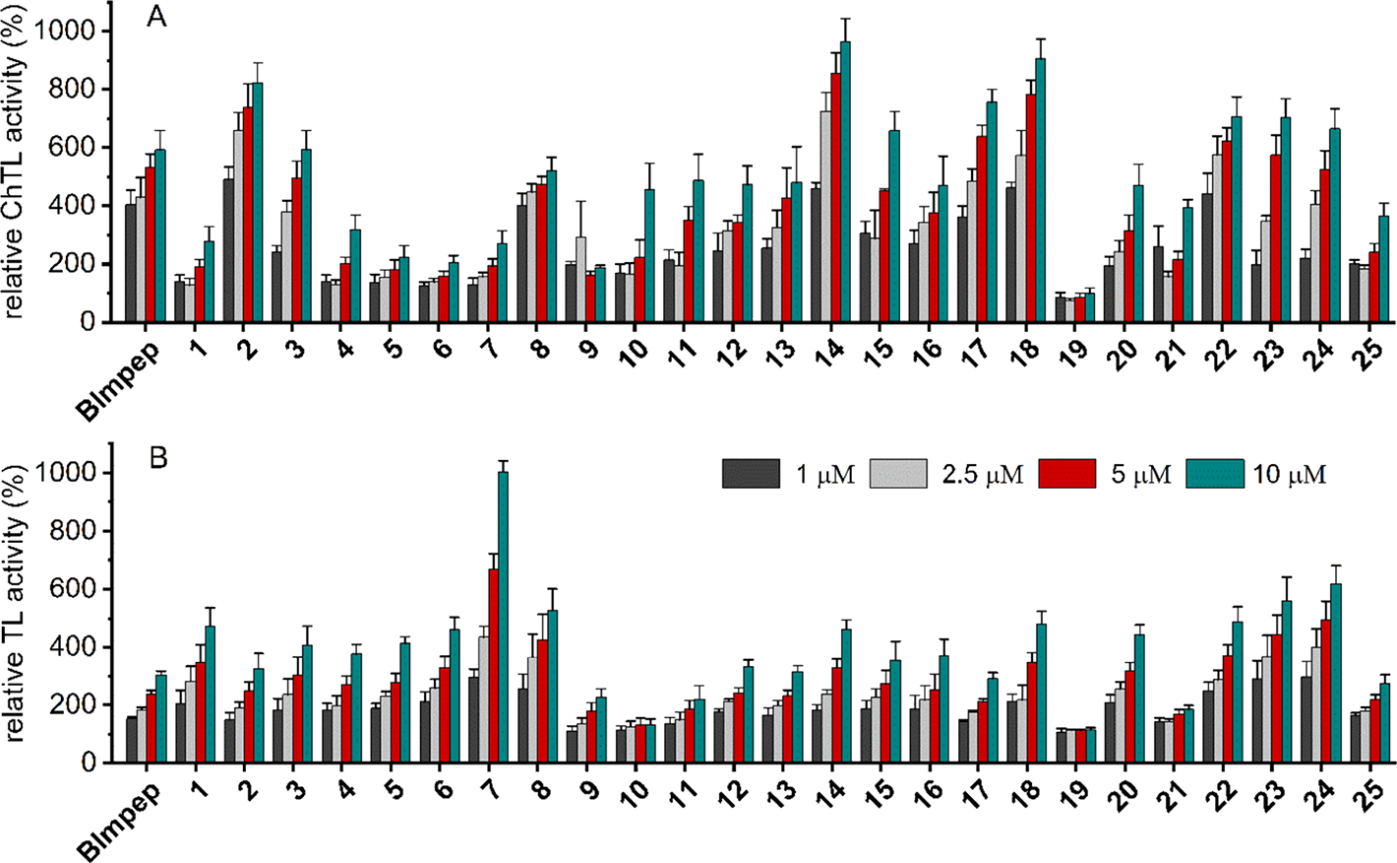
Comparison of the influence of Blm modulators on (A) the ChT-L peptidase (probed with Suc-LLVY-AMC) and (B) the T-L peptidase (probed with Boc-LRR-AMC) of the latent human 20S proteasome. Results were calculated in relation to the latent h20S (control), whose activity was regarded as 100%. The results are the means of at least three independent experiments performed in two technical replicates. The variability of each data point was presented as standard deviation (SD).

The stimulating capacity of the designed modulators was probed using classic fluorogenic substrates: Suc-LLVY-AMC for the chymotrypsin-like, Boc-LRR-AMC for the trypsin-like, and Z-LLE-AMC for the caspase-like activity. The ChT-L and T-L peptidases did not respond in the same way to the presence of modulators. The most striking examples are compounds **2**, **14**, and **18**, which were clearly superior to other modulators in stimulating the ChT-L activity (Figure 1A), but did not show such capacity for the T-L (Figure 1B). On the other hand, compound **7,** which was the most active stimulant of the T-L peptidase, was concurrently among the least effective ones against the ChT-L.

Various polar residues were introduced at positions 5 and 6 to enable interactions with polar patches adjacent to the binding pockets. These changes had a beneficial effect on the ChT-L activity only in the combination of a polar residue at position 5 and a basic residue at position 6 (Figure 1A, compound **2**). The acidic substitutions in the 5–6 region failed to provide interactions profitable for the ChT-L peptidase. Moreover, the acidity of this region was counterproductive and resulted in a significant decrease in the stimulating capacity (Figure 1A, compounds **4**–**7**). Basic residues, such as Lys6 in **2** and **24** and Arg5 in **8** and **22**, were more preferred; nevertheless, only **2** was an activator superior to Blm-pep. Compound **2** activated the enzyme almost 1.5 times more effectively than Blm-pep, and at a concentration of 1 μM, it accelerated the degradation of Suc-LLVY-AMC 8-fold in comparison to the control. Both **2** and **3** were much more effective than **1** in stimulating ChT-L activity. The main difference between these compounds is the Lys1 residue, present in **2** and **3**, but not in **1**. This result demonstrated the importance of a positive charge at the modulator’s *N*-terminus.

It is interesting that the effectiveness and specificity of modulators can be influenced by modifying both their *C*-terminal and *N*-terminal sequences, despite the fact that the latter fragment was invisible in the crystal structure of the y20S-Blm-pep complex. Although the lack of electron density could suggest negligible interactions and therefore the total lack of significance of the *N*-terminal region, our findings demonstrated that this is not true. In some cases, even small changes in the N-terminal sequence contributed to variations in modulators’ efficiency and specificity. Good examples are compounds **6** and **7**, in which residues with the same acidic character were introduced as modifications to positions 1, 5, and 6, but while compound **6** had Asp, Glu, and Asp in these positions, compound **7** had Glu, Asp, and Glu, respectively. This change did not differentiate the ability of the compounds to stimulate the ChT-L peptidase (Figure 1A), but it had a large effect on the T-L (Figure 1B). The similar pattern of stimulation and lack of such capacity toward the T-L and ChT-L active sites, respectively, was observed for the natural allosteric activator PA200, the human ortholog of Blm10.^13^ The resolved cryo-EM structure of the PA200-proteasome complex revealed that upon binding of the activator, the S1 pocket of the β5 became narrower, while the one in the β2 subunit widened. It was hypothesized that this change facilitated the accommodation of a bulky Arg residue, thereby improving the T-L performance. The interactions provided by the *N*-terminal region of compound **7** may similarly, through the propagation of allosteric signals, affect the direct neighborhood of the active sites.

Optimization of the *C*-terminal sequence was composed of introducing a hydroxyl group into the side chain of the ultimate residue to provide interactions that could more strongly anchor a modulator within the α pocket. Additionally, to further strengthen the interactions, changes were introduced in the region encompassing residues 8–11. The incorporation of an acidic residue in this region was very beneficial, but only in the DW/DY/EW arrangement, not in the one reversing the order of acidic and aromatic residues. Compound **14**, with the DY sequence, was about two times more effective in stimulating the ChT-L peptidase than **13**, in which the same amino acids were arranged in reverse order (Figure 1A). Of importance was also the basicity of a residue at position 11. Compound **15**, which has the DYRS sequence in the 8–11 region, was less effective in activating the ChT-L peptidase than **14** with the DYRR segment in the same place.

Combining changes in the *N*-terminal region of Blm-pep with the most profitable modifications of its *C*-terminal part yielded effective activators (compounds **22**, **23**, and **24**) but did not increase their ability to stimulate the ChT-L peptidase to levels higher than the parent peptide. The best activators of ChT-L were **14**, **17**, and **18**, in which the Blm-pep sequence has only been modified on the *C*-terminal side. The effectiveness of compounds **17** and **18** in stimulating the degradation of Suc-LLVY-AMC was confirmed in tests that investigated the kinetics of the enzymatic reaction (Figures S3 and S4). EC50 values for these compounds were 3.52 ± 0.28 and 3.49 ± 0.36 μM, respectively. Combining changes in the modulators’ *N-* and *C-*terminal segments was more profitable for the activation of the T-L peptidase. Compound **24**, designed as an assembly of the *N*- and *C*-terminal sequences of **2** and **18**, respectively, surpassed not only Blm-pep but also both parent peptides in the ability for stimulating the proteasome to degrade the Boc-LRR-AMC substrate (Figure 1B). In the case of the C-L peptidase, **24** was still a better activator than **2** but did not display higher activity than **18** (Figure S5).

### Mechanism of Action of Blm-pep Analogs

**Figure 2:**
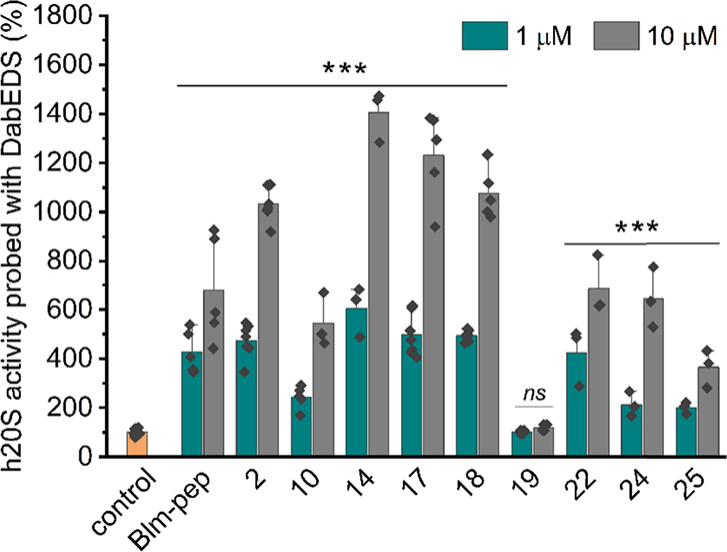
Effect of Blm modulators on the degradation of FRET-type polypeptide substrate, DabEDS, by h20S. Results were calculated in relation to the latent h20S (control), whose activity was regarded as 100%. The results are the mean of at least three independent experiments performed in two technical replicates. Standard deviation was displayed for each activity data. One-way ANOVA with Tukey’s post hoc test was used to determine statistical significance of the observed differences in relation to the control (***p < 0.001; ns, statistically nonsignificant).

Due to their small size, classical fluorogenic substrates can quite easily pass through the gate and be digested even by the latent 20S. To confirm the stimulating capacity of the selected compounds, we performed assays using a FRET-type substrate with a peptide chain elongated to 11 residues (Lys(Dabcyl)-Met-Ser-Gly-Phe-Ala-Ala-Thr-Ala-Glu(EDANS)-Gly; hereinafter abbreviated as DabEDS).^35^ This substrate is very poorly digested by the latent 20S proteasome, which allowed us to obtain results clearly separated from the control, with differences between the individual compounds being more clearly pronounced (Figure 2, Figures S6 and S7). As we verified by mass spectrometry, DabEDS is digested by the proteasome after Lys, Glu, and Gly residues, thus different active sites are likely involved in its proteolytic degradation. Therefore, this longer substrate better illustrates the action of the proteasome during protein digestion, when the active sites act cooperatively. Experiments with DabEDS confirm the very high stimulating capacity of **14**, **17,** and **18** (Figure 2). EC50s determined using this substrate are 3.21 ± 0.78 and 2.64 ± 0.60 μM for compounds **17** and **18**, respectively. Analog **19** differs from one of the best activators, compound **18**, only by a single residue. Although molecular modeling suggested the Tyr13Asp exchange as beneficial, it transformed one of the most effective activators into a compound completely devoid of stimulatory capacity (Figures 1 and 2 and Figures S6 and S7). This result indicates that Blm modulators act similarly to their parent activator Blm10, which requires the intact HbYX motif for allosteric activation of the proteasome.

A thioflavin T assay proved that the observed differences in stimulating capacity were not due to aggregation of the modulators (Figure.S8). Analyzing by HPLC the stability of the modulators in the presence of h20S proteasome, we also excluded the possibility that the lower stimulating capacity is due to increased competition with the substrate. If the action of the modulators was based solely on competition, the compounds degraded to a greater extent would impede the access of a fluorogenic substrate to the proteolytic site, resulting in diminished fluorescence. The effect that we observed, however, was exactly the opposite. The yield of proteolysis was directly correlated with the activating effect: compounds **2**, **14**, **17**, and **18**, which are the most effective activators, were digested the most rapidly (Figure S9). Compound **19**, which was unable to stimulate any peptidase, was resistant to proteolytic degradation. This provides indirect evidence for an allosteric mechanism of action of Blm peptides, analogous to that of the 19S or PA200 activators. This action probably loosens the conformation of the gate leading to the catalytic channel, increasing the dynamics of its alternation between the closed and open states, thereby allowing the entry and degradation of both substrates and modulators.

**Figure 3:**
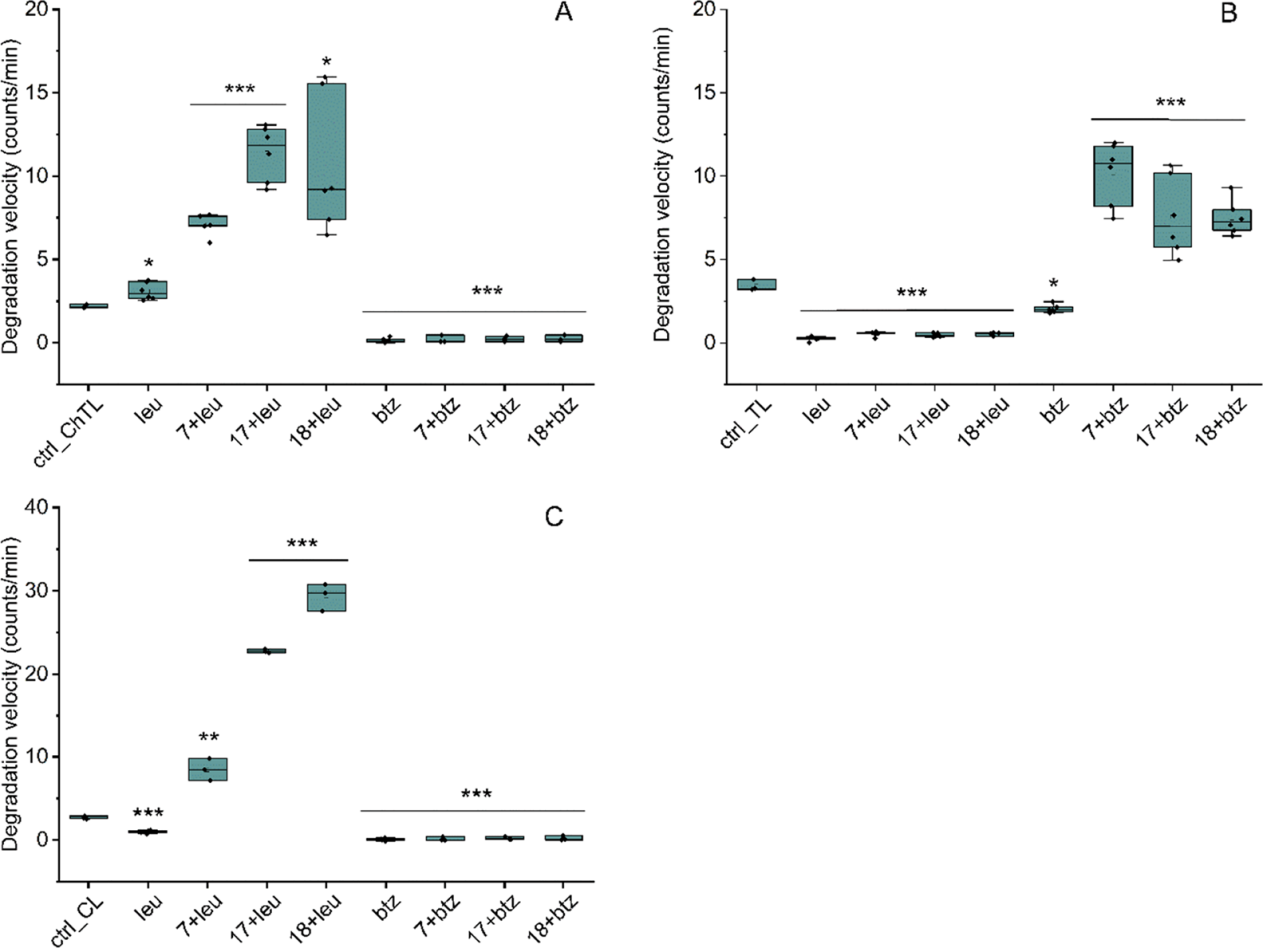
Velocity of the substrate degradation by the h20S inhibited with either 50 μM leupeptin (leu; a specific inhibitor of β2) or 10 μM bortezomib (btz; a specific inhibitor of β5) in the absence and presence of Blm analogs, **7**, **17**, or **18**. The concentration of the activators was 10 μM. Proteasome activity was probed using the specific substrate of (A) ChT-L peptidase, Suc-LLVY-AMC (100 μM); (B) T-L peptidase, Boc-LRR-AMC (100 μM); (C) C-L peptidase, Z-LLE-AMC (20 μM). Results are the means of at least three independent experiments performed in two technical replicates. Standard deviation was displayed for each data. One-way ANOVA was performed and confirmed statistical significance of the observed differences between the control and each data (*p < 0.05; **p < 0.01; ***p < 0.001).

There are several hypotheses regarding the details of the effect of allosteric activators on proteasome activity. The most general hypothesis is that activators binding to the α ring increases the dynamics of conformational changes and causes a wider opening of the proteasome gate.^11,14,37^ Another mechanism has been proposed based on the cryo-EM structure of the 20S-PA200 complex.^13^ Toste-Rêgo and da Fonseca interpreted the observed change in the size and electrostatic potential of the substrate pocket in the β5 subunit as evidence that its specificity was converted from ChT-L to T-L, which they considered responsible for the increase in the T-L peptidase activity. Yet, another potential mechanism was recognized by Thomas and Smith.^38^ Based on the studies with selective inhibitors of the ChT-L and T-L peptidases, they proved that the PA28γ activator upregulates the T-L activity through allosteric signal transduction directly to the T-L active site. To check what effect blocking binding sites would have on the performance of Blm modulators, we conducted similar experiments using bortezomib and leupeptin, which preferentially inhibit the β5 and β2 active sites, respectively. In tests with the Suc-LLVY-AMC substrate, we noted a complete loss of proteasome activity after treatment with bortezomib and the activators were not able to overcome the inhibition (Figure 3A). In the presence of leupeptin, the ChT-L substrate was still digested and the activators increased the rate of degradation. The corresponding effect was observed in tests with the Boc-LRR-AMC substrate (Figure 3B). These results indicate that the activators are able to induce the opening of the gate leading to the catalytic compartment, and enhance the rate of substrate degradation even when an inhibitor is present in the active site provided that it is not the active site specific for this substrate. Furthermore, the experiments with inhibitors allowed for confirmation of the specificity of the activators, as compound **7** was more effective in stimulating the T-L peptidase, while **17** and **18** were more effective in stimulating the ChT-L. For the C-L peptidase, the relationship between the type of inhibitor and the ability of modulators to overcome its inhibitory effect was similar to that observed for the ChT-L (Figure 3C). This diversity suggests that Blm activators may not only affect the gate state but also modulate the efficiency of individual active sites.

### Influence of Modulators on the Degradation of Native Proteins

**Figure 4:**
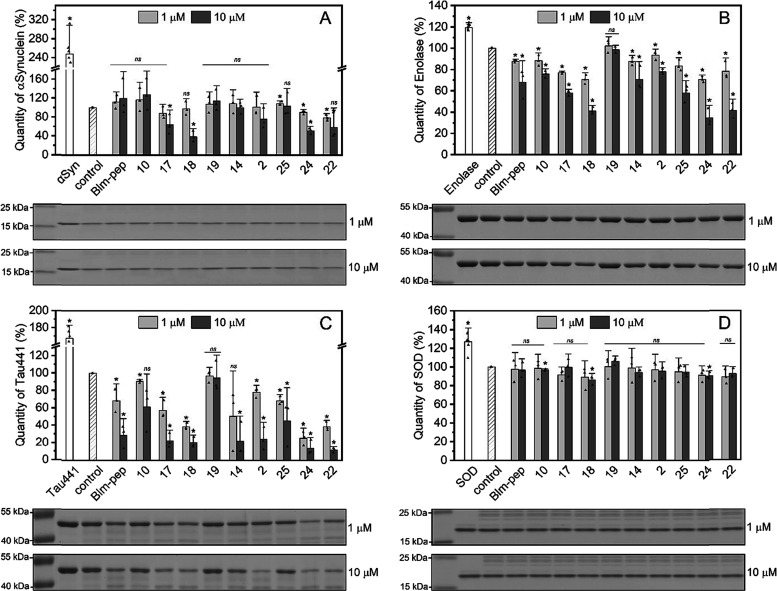
Effect of Blm modulators on the digestion of native proteins: (A) α-synuclein, (B) enolase 2, (C) Tau protein, and (D) superoxide dismutase 1 (SOD1). The proteins were incubated with proteasome for the time allowing us to observe changes in the rate of protein digestion by the proteasome alone and in the presence of modulators (from 1.5 h for native α-synuclein to 21 h for SOD). The graphs depict the relative amount of each protein that remained after its incubation with h20S in the absence (control) and the presence of modulators at a concentration of either 1 or 10 μM. Below the graphs, representative electropherograms are shown, separately for 1 and 10 μM modulator concentrations. On each of the electropherograms, the leftmost there are marker bands. Next to them are bands corresponding consecutively to the protein in the absence of h20S, in the presence of h20S (control), and in the presence of h20S and modulators. The modulators on the electropherograms are in the same order as those on the graphs. Experiments were performed in three biological replicates. Error bars represent the standard deviation. Statistical analysis was performed using ANOVA with a post hoc Bonferroni test, and indicated which results have statistical significance in relation to the control. A *p* value less than or equal to 0.05 indicates a statistically significant result (*); ns means “statistically nonsignificant”.

The effectiveness of the compounds has been examined also using proteins as substrates. Utilizing SDS-PAGE, we checked the extent to which model proteins were digested by 20S proteasome alone and after its stimulation by the modulators, at 1 and 10 μM concentrations. Human native α-synuclein, which is a relatively small (120aa) and mainly unstructured protein, was degraded in 60% after 1.5 h of incubation both in the absence and in the presence of 1 μM modulators. However, a 10-fold higher concentration of the modulators, especially **17**, **18**, **22**, and **24**, significantly increased the rate of synuclein proteolysis (Figure 4A). Human enolase, which is composed of 434 amino acid residues and has many regions with a strictly defined secondary structure, was proteolyzed to a much lower extent. 3 h of incubation with h20S left about 80% of the protein undigested. Remarkably, this protein also was degraded efficiently in the presence of **17**, **18**, **22**, and **24**. At 10 μM concentrations of **18**, **22**, and **24**, the increase in the efficiency of degradation was more than twofold (Figure 4B).

Since the digestion of abnormal or overexpressed proteins is particularly important in the context of neurodegenerative diseases, we performed also experiments assessing the h20S capacity of degrading Tau protein, associated with the development of Alzheimer’s disease, and superoxide dismutase, a mutation of which is connected with the development of amyotrophic lateral sclerosis. The enhanced proteolysis of Tau-441 was observed for all tested compounds except for **19** (Figure 4C). The protein was degraded with 40% efficiency by 20S alone whereas in the presence of already 1 μM modulators, the digestion rate increased by as much as 25–75%, depending on the modulator. More spectacular acceleration of the Tau-441 digestion was observed when the modulators were used at 10 μM concentration. In the case of the second model protein, native superoxide dismutase, none of the modulators was able to significantly affect the efficiency of proteolysis (Figure 4D).

### Determination of the Modulator’s Influence on the Levels of Oxidized Proteins

The primary role of the 20S core particle is to digest abnormal proteins.^39^ The amount of such proteins in cells increases, for instance, under conditions of oxidative stress.^40^ Oxidized proteins cannot fulfill their function; therefore, they become redundant. More importantly, they usually lose their native conformation, which increases their tendency to oligomerize and aggregate.^41^ To prevent such a scenario, proteolysis of aggregation-prone biomolecules must be intensified. Therefore, we checked whether the modulators we designed would be able to stimulate the 20S proteasome to more intensively degrade oxidized versions of the model proteins, α-synuclein, and enolase.

**Figure 5:**
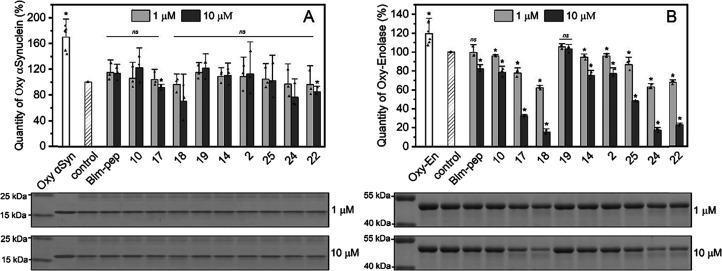
Effect of Blm modulators on the digestion of (A) oxidized α-synuclein and (B) oxidized enolase 2. The graphs show the relative amount of each protein that remained after its incubation with h20S in the absence (control) and the presence of modulators at 1 or 10 μM concentration. Below the graphs, representative electropherograms are shown, separately for 1 and 10 μM modulator concentrations. On each of the electropherograms, the leftmost there are marker bands. Next to them are bands corresponding consecutively to the protein in the absence of h20S, in the presence of h20S (control), and in the presence of h20S and modulators. The modulators on the electropherograms are in the same order as on the graphs. The samples were incubated at 37 °C for 3 h. Experiments were performed in three biological replicates. Error bars represent standard deviation. Statistical analysis was performed using ANOVA with a post hoc Bonferroni test and indicated which the results have statistical significance in relation to the control. A *p* value less than or equal to 0.05 indicates a statistically significant result (*); ns means statistically nonsignificant.

The experiments confirmed the literature reports that oxidatively modified synuclein is less efficiently digested by proteasome than the native one.^42^ Even after 3 h of incubation, h20S was unable to digest about 60% of the oxidized synuclein (Figure 5A), whereas only 40% of the native variant was left undigested in two-times shorter experiments (Figure 4A). The modulators were unable to considerably increase the rate of degradation of the oxidized version of α-synuclein. In the case of enolase, both the native and oxidized proteins were degraded weakly by the 20S alone; however, the presence of modulators, especially at 10 μM concentration, enhanced the proteolysis (Figures 4B and 5B). Interestingly, a more pronounced effect was observed for the oxidized protein. The stronger effect of Blm-pep analogs on the degradation of oxidized enolase is a desirable result as it means that it would be possible to selectively remove defective proteins in the presence of their natively folded forms.

### Activating Potential of Blm Analogs in Cell Lysate and in Intact Cells

**Figure 6:**
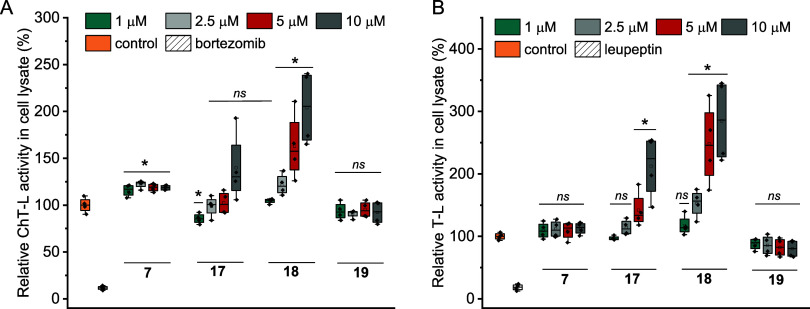
Influence of the selected Blm modulators on the degradation of substrates (A) Suc-LLVY-AMC and (B) Boc-LRR-AMC, in the HEK293T cell lysate. Results are the means of two biological replicates performed in two technical repeats. The control is the cell lysate treated with a vehicle. The proteasome inhibitors, 10 μM bortezomib (preferentially inhibiting the β5 subunit, which is responsible for the ChT-L activity) and 50 μM leupeptin (selective to the β2 subunit, which is responsible for the T-L activity), were negative controls. The variability of each data was presented as standard deviation. One-way ANOVA indicated which results have statistical significance in relation to the control (* *p* < 0.05; ns, statistically nonsignificant).

To assess whether Blm analogs are able to stimulate the proteasome in the cellular environment, we conducted experiments using human embryonic kidney (HEK) 293T cell lysate. For these studies, we selected the compounds that proved to be the most effective in tests on the isolated 20S proteasome. For comparison, compound **19**, which had no effect on any of the proteasome’s peptidases, was also included. The results confirmed the stimulating potential of Blm analogs. Compounds **17** and **18** seem very promising since at 10 μM concentration, they were able to increase the proteasome activity by about 1.5–2.5-fold (Figure 6).

**Figure 7:**
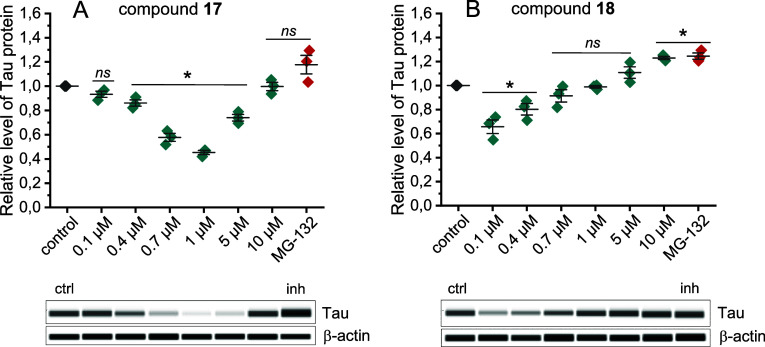
Relative level of Tau, fused to GFP, in intact HEK293T cells after treatment with compound (A) **17** or (B) **18**, for 24 h. The protein level was detected by Western blot analysis using an automatic capillary WES platform and specific anti-GFP antibodies. Representative Western blots are shown below each chart. Concentrations of modulators **17** and **18** in each blot are shown above the corresponding lanes. MG-132 proteasome inhibitor was used at a final concentration of 10 μM. Experiments were performed in three biological replicates. Error bars represent standard deviation. Statistical analysis was performed in relation to the control using ANOVA test with a significance level of *p* < 0.05 (*); ns, statistically nonsignificant.

We also investigated how modulators **17** and **18** act in intact HEK293T cells. We tested the influence of these compounds on the degradation of Tau and mutated SOD1 (SOD1G37R), both fused with the GFP reporter protein. The proteins were overproduced after transfection of the cells with plasmids bearing genes which encode them. The tested compounds were added to cell cultures and entered the cells during the transfection procedure. Western blot analysis of the protein levels in the cells overproducing AD-related Tau showed that both **17** and **18** enhanced Tau degradation, but compound **17** exerted a stronger effect (Figure 7). Interestingly, at higher concentrations, both peptides showed decreasing ability to stimulate Tau proteolysis.

**Figure 8:**
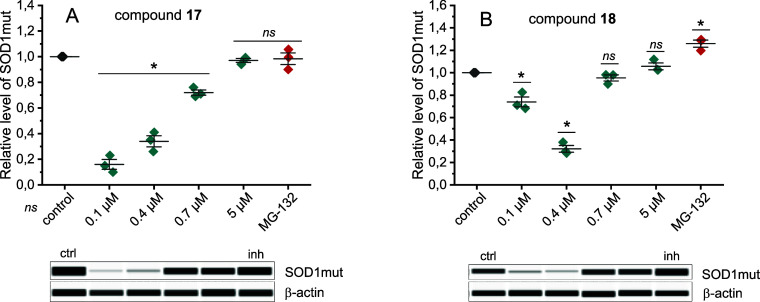
Relative level of mutated SOD1 (SOD1G37R), fused to GFP, in intact HEK293T cells after either **17** (A) or **18** (B) treatment for 24 h. The protein level was detected by Western blot analysis using an automatic capillary WES platform and specific anti-GFP antibodies. Representative Western blot is shown below each chart. MG-132 proteasome inhibitor was used at a final concentration of 10 μM. Experiments were performed in three biological replicates. Error bars represent standard deviation. Statistical analysis was performed in relation to the control using ANOVA test with a significance level of *p* < 0.05 (*); ns means statistically nonsignificant.

Similar and even more pronounced effects were observed in the cells overproducing the mutant SOD1 that is related to the development of amyotrophic lateral sclerosis.^45^ Both modulators at concentrations of 0.1 and 0.4 μM significantly accelerated the degradation of the protein (Figure 8). In addition, compound **17** proved to be a more effective stimulator, in the presence of which no more than 20% of mutant SOD1 remained compared to the control. This result is particularly interesting since it is believed that the pathogenicity of SOD1 mutations is not due to a lack of the functional protein but rather to the accumulation of its misfolded and aggregated forms.^46^

As with Tau, SOD1G37R degradation was effectively stimulated by low concentrations of the modulators. For higher concentrations, the effect disappeared. We tested various hypotheses that could explain the observed peculiarity. First, we ruled out that the loss of the ability to stimulate the digestion of fusion proteins was due to aggregation of the modulators, since they did not show such propensity (Figure S8). The second hypothesis considered the fact that under cellular conditions, the 20S catalytic core is not the only possible form of proteasome. Along with it, protein digestion is carried out by the 26S complex. It has one or two copies of the 19S regulator attached to the α rings of the core particle. The 19S recognizes proteins destined for degradation and unfolds them, allowing large molecules to translocate into the catalytic chamber. These processes require ATP, which is a very unstable compound rapidly hydrolyzing during cell lysis. Hence, 26S can significantly participate in proteolysis only in intact cells but not in cell lysate unless replenished with ATP. Since the vanishing stimulatory effect of the increasing modulators’ concentration was observed in intact cells only, it seemed likely to be related to the 26S proteasome. We supposed that modulators at higher concentrations might interact with 26S at additional sites that exhibit a weaker apparent binding constant and negative cooperativity with the main binding site. Therefore, we investigated whether compounds **17** and **18** can exert any effect on the human 26S proteasome. The experiments were performed on the isolated enzyme (Enzo Life Sciences, Farmingdale, NY, USA) and allowed us to establish that neither **17** nor **18** influences the activity of h26S (Figure S12). Thus, we hypothesize that the loss of the ability to intensify the degradation of model proteins may be due to the binding of modulators present in excess to GFP-Tau and GFP-SOD1 constructs and hindering their proteolysis. A possible explanation is also the overall effect on cellular metabolism in a way that impairs the function of the 20S proteasome. Further study of these mechanisms is needed, but this is beyond the scope of this work. Nevertheless, the ability of proteasome activators at low concentrations to boost removal of aggregation-prone proteins is a very promising result. It encourages further studies of Blm activators to develop analogs that have more drug-like properties. Such studies are ongoing.

### Activator 18 Binds in the Pockets between the α-subunits

**Figure 9:**
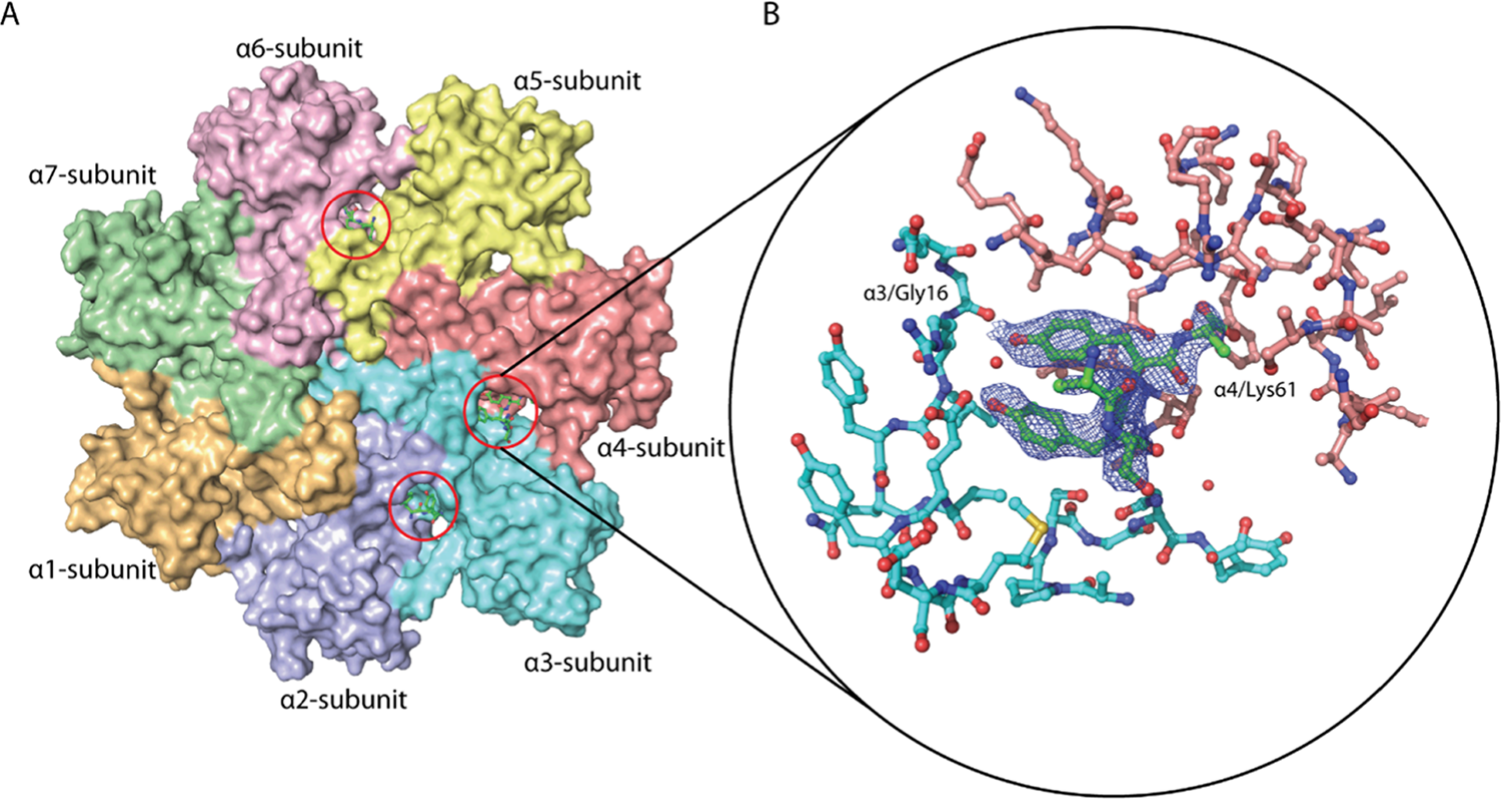
(A) The binding sites of compound **18** at the α-subunits of h20S are shown as surface representation. (B) Enlarged view of the modulator (green) inside the binding pocket. The penultimate tyrosine’s hydroxyl group creates a hydrogen bond with the backbone carbonyl of Gly16, and the *C*-terminal carboxylic group of the modulator is directed toward the conserved Lys61 residue. The 2fo-fc density (blue mesh) is contoured at 1 σ. Protein Data Bank accession code 8BZL.

We crystallized the human 20S proteasome and soaked the crystals with **18** (Table S1). In the resolved structure, the modulator was observed in three pockets: α2-α3, α3-α4, and α5-α6, on both the α rings (Figure 9). In the α3-α4 pockets, five *C*-terminal amino acid residues (10–14) of **18** can be fitted to the electron density, whereas only three to four residues can be modeled into the electron density present in pockets α2-α3 and α5-α6. Some side chains of the modulator’s residues could not be resolved in the electron density. The crystal packing and restricted conformational space in the crystal lattice might explain the differences in the observed electron densities in the pockets. Compound **18** penetrates deeply into each pocket and interacts with the proteasome through a set of contacts typical for activators containing the HbYX motif. The *C*-terminal carboxylic group of the modulator is directed toward the conserved Lys residue located at the bottom of the pockets (α3Lys64, α4Lys61, and α6Lys62). The hydroxyl group of the penultimate tyrosine creates a hydrogen bond with the backbone carbonyl of Gly (α2Gly16, α3Gly16, and α5Gly19).

**Figure 10:**
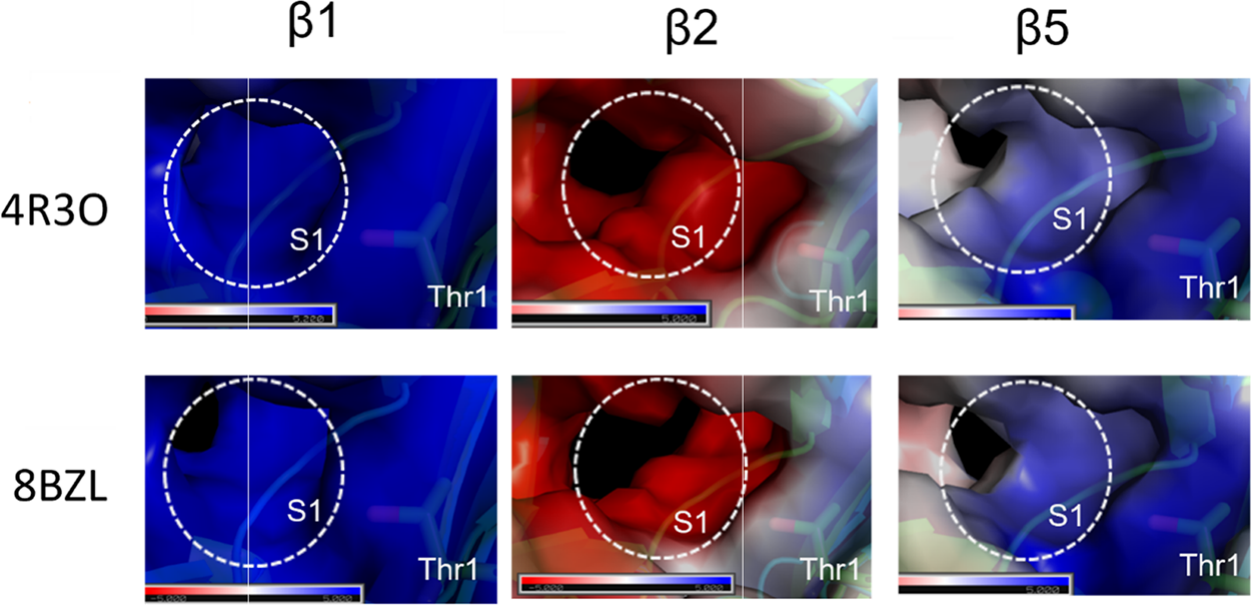
Comparison of the proteolytic sites in the human 20S proteasome (4R3O) and the 20S-**18** complex (8BZL). Shown are van der Waals surface representations of the three proteasome active sites (β1, β2, and β5), viewed from the proteasome inner cavity. The surface is colored by charge (blue—positive, red—negative, white—neutral).

Despite multiple binding sites occupied by **18,** the gate leading to the catalytic channel remains closed in the resolved structure. As demonstrated by the cryo-EM structures of the 19S-20S complex, docking of the *C*-terminal HbYX motifs in even 3 pockets between the α subunits has not yet led to the permanent opening of the proteasomal gate.^14,31^ On the other hand, the monomeric activators Blm10 and PA200, which dock the HbYX motif in only one, the α5-α6 pocket, cause significant conformational changes and partial opening of the entrance to the catalytic channel.^13,29^ However, as demonstrated for PA200, even a relatively wide gate opening does not cause activation of all peptidases, but affects the selected active sites and changes their substrate preference.^13^ We therefore tested whether the binding of **18** induces any changes in the S1 substrate pockets. As for the 20S-PA200 complex, we observed the pocket widening, but not only in the β5 subunit but also in β1 and β2 (Figure 10). To some extent, the electrostatic potential in the β5 pocket also changed. Thus, it seems that the allosteric mode of action in some cases causes stable and permanent opening of the entrance to the catalytic chamber, but in others, it merely shifts the dynamic equilibrium toward a looser gate conformation, allowing it to open more frequently, albeit only transiently. However, in both cases, the conformational changes can propagate to the active sites, stimulating proteasome activity.

## Discussion

In recent years, peptides have attracted considerable interest as molecules that combine the advantages of protein and small-molecule therapeutics. A total of 33 peptide drugs have been approved worldwide since 2000 and more than 170 peptides are in active clinical development.^47^ Peptides, like proteins, have a significant affinity for their targets, which makes them effective and specific. At the same time, they are less immunized and less expensive to produce than proteins. In addition, they tend to exhibit less toxicity than low-molecular-weight compounds. On the other hand, however, peptides have difficulty penetrating biological membranes and often have insufficient proteolytic stability. To become a drug, they must be modified by attaching a cell-penetrating sequence and replacing native amino acids with their unnatural counterparts. Nevertheless, even simple peptides are invaluable tools in identifying target requirements and facilitating the design of therapeutics.

Designing activators of the proteasome is challenging, because it is a large multimeric protein with numerous potential sites on its surface that can bind modulators. Moreover, the active sites are deeply hidden, and the pathway by which the signal is transmitted from the binding site to the catalytic channel is unknown. Furthermore, the proteasome has active sites with three different specificities that can respond differently to the transmitted allosteric signal. Gathering as much knowledge as possible about the binding sites of synthetic modulators of the proteasome, their mechanism of action, and the structure–activity relationship is necessary to enable the design of drugs for diseases associated with inefficient proteolysis carried out by the 20S proteasome.

Utilizing the *C*-terminal sequence of Blm10 and molecular modeling, we designed 25 peptides and examined their ability to activate the human 20S proteasome. In the presence of the best activators, at 10 μM concentration, the proteasome was 8–10 times more effective in degrading low-molecular-weight substrates of the ChT-L and T-L peptidases (Figure 1) and several times more efficient in the proteolysis of the FRET-type polypeptide substrate (Figure 2). The activators most efficiently affecting the ChT-L peptidase had an acidic residue at position 8 in combination with an aromatic residue at position 9. The presence of a basic residue at the N-terminus is also important. Replacing it with an acidic residue clearly reduced the activation capacity (Figure 1A, **18** versus **20**). The opposite effect was observed for the T-L peptidase, where an acidic residue was preferred at the N-terminus. For this peptidase, positions 5 and 6 were also important, where acidic residues of an appropriate size should be present. These results indicate that modifications introduced in the N-terminal region, although this region is not visible in the crystal structures of proteasome-modulator complexes (PDB: 8BZL, 5NIF, 4X6Z), affect not only the efficiency but also the specificity of the activators.

In the crystal structure of proteasome with one of the best activators, compound **18**, the modulator’s residues were observed in three α pockets. This demonstrates that our efforts to design the activator sequence in such a way that it can bind in more than one pocket and thus better mimic a multivalent protein activator have yielded positive results. Nevertheless, we were unable to observe the opening of the gate leading to the catalytic channel. This resembles the situation that was detected with the 19S activator. Cryogenic electron microscopy structures proved that placing even three the HbYX motif-bearing C-terminals of 19S in the pockets between α-subunits does not lead to an open gate.^48^ Additional interactions, including those of the non-HbYX-terminated subunit, were required to yield a stable open conformation. Since modulator **18** binds only in three pockets, this probably is not sufficient to allow stabilization of the open form. It has been reported that the latent 20S alternates between the open and closed states, with about 25% of its molecules having an open gate and 75% a closed gate.^15^ Thus, we speculate that the activating effect of Blm modulators is related to relaxing the proteasome’s conformation and increasing the dynamics of transitions between the open and closed structures, facilitating the entry of substrates into the catalytic channel.

However, our research indicates that the action of modulators is not limited to affecting the gate state. The crystal structure has shown that Blm activators can transmit allosteric signals to the catalytic sites and induce changes in the substrate pockets (Figure 10). The effect beyond the gating mechanism is also implied by variation in the ability of modulators to stimulate individual peptidases in the presence of inhibitors. Bortezomib completely blocked the activity of the ChT-L and C-L and, as expected, prevented the degradation of the substrates of these peptidases even in the presence of modulators (Figure 3). In turn, modulators **17** and **18** were able to stimulate both peptidases of the leupeptin-treated proteasome. Moreover, they were clearly more efficient than compound **7**. In contrast, in the presence of bortezomib, which, unlike leupeptin, does not block the active site responsible for T-L activity, compound **7** worked more efficiently than **17** or **18**.

The ability of Blm modulators to activate the human 20S proteasome was also confirmed in tests with protein substrates. The modulators stimulated the degradation of both native and oxidized enolase as well as synuclein and Tau proteins associated with neurodegenerative diseases. Digestion of proteins by the 20S proteasome was proposed to be mediated through contacts which the protein hydrophobic patches made with the enzyme’s α-surface.^39^ It was hypothesized that these contacts could cause the opening of the catalytic channel and stimulate the proteolysis. Oxidation of folded proteins can lead to greater exposure of such hydrophobic regions, facilitating their interaction with the proteasome and digestion of proteins. However, unfolded proteins can undergo oligomerization, and oligomers can inhibit proteasome.^49^ Small-molecule activators could be a remedy for such a situation, as they can restore the proteolysis carried out by the 20S core particle. As our results indicated, these modulators can even provide a certain degree of selectivity. Blm analogs, although all based on the same HbYX binding motif, show a diverse capacity of stimulating the degradation of different biomolecules.

In conclusion, our research has provided information relevant to the design of effective and selective modulators of the 20S proteasome. Further experimental work aimed at obtaining proteolytically stable compounds capable of penetrating cells is currently in progress.

## Materials and Methods

### General Information

All reagents and solvents used for sample preparation were of molecular biology grade and were used as purchased without further purification. The pH of all buffers was determined at 20 °C. 20S proteasome was isolated from human erythrocytes and purified as it was previously described.^50^

#### Molecular Modeling

The following crystal structures of proteasome were used for the modeling process: human constitutive 20S proteasome (PDB ID: 4R3O), yeast 20S proteasome in complex with Blm10 (PDB ID: 4 V7O), and yeast 20S proteasome in complex with Blm-pep (PDB ID: 4ZZG). In the 4ZZG structure, electron density was observed only for the fragment corresponding to the five *C*-terminal amino acid residues of the activator. This fragment was modeled sequentially into every α pocket of the human proteasome. One substitution in comparison to the original Blm-pep sequence was made: alanine was placed in position 5 instead of glycine to get information on possible contacts of this residue. The newly obtained complexes were optimized with the Amber ff14SB force field.^51^ Low-temperature molecular dynamics and structure minimization in repetitive cycles were used to maintain the position of the ligand. The obtained results were visualized by using the UCSF Chimera and PyMol program. The electrostatic surface was calculated using APBS, the adaptive Poisson–Boltzmann solver [10.1002/pro.3280] implemented in the PyMol. Two grooves were selected around each pocket as potential interaction sites for the *N*-terminal part of the activators. The models were analyzed using UCSF Chimera, PyMol, and LigPlot programs. Based on this analysis, amino acid residues which could provide profitable interactions in each direction were proposed.

#### Peptide Synthesis

Syntheses of all peptides were carried out on a solid support (Wang or Cl-TCP (Cl) Protide resin) using a Liberty Blue microwave peptide synthesizer (CEM). Coupling of Fmoc-protected amino acid residues was carried out using 1 M diisopropylcarbodiimide/0.5 M ethyl cyano(hydroxyimino)acetate 1:1 mixture. Crude peptides were purified by reverse-phase high-performance liquid chromatography (RP-HPLC), using a C12 semipreparative Jupiter Proteo column (21.2 × 250 mm, 4 μm, Phenomenex) and linear gradients of acetonitrile in 0.1% TFA in water. The purity of the peptides was assessed by analytical HPLC using a Luna C18 column (4.6 mm × 250 mm, 5 μm, 100 Å; Phenomenex) and an LC-20A HPLC system (Shimadzu). Quantitative analysis was performed based on the integration of the area under the peaks, using the Lab Solution software provided by the HPLC manufacturer (Shimadzu). The purities of all final compounds were 95% or higher. The identity of the pure products was confirmed based on *m*/*z* signals detected by a LCMS-ESI-IT-TOF Prominence mass spectrometer (Shimadzu).

#### Enzymatic Activity Tests

The capacity of influencing the human 20S proteasome was examined using the latent enzyme isolated from human erythrocytes. Boc-LRR-AMC, Z-LLE-AMC, and Suc-LLVY-AMC fluorogenic substrates (Enzo Life Sciences Inc.) were used for assessment of the trypsin-, caspase-, and chymotrypsin-like activities, respectively. As an additional probe, we utilized a homemade FRET-type substrate DabEDS (Lys(Dabcyl)-Met-Ser-Gly-Phe-Ala-Ala-Thr-Ala-Glu(EDANS)-Gly^36^). Stock solutions of the substrates and modulators were prepared in dimethyl sulfoxide (DMSO). The final concentration of the organic solvent did not exceed 0.02%. The activity assays were performed in a 96-well plate format in 50 mM Tris/HCl, pH 8.0. The CP was used at a final concentration of 0.002 mg mL^–1^ (2.8 nM). The final concentrations of LLVY, LRR, and LLE substrates were 100 μM and DabEDS 15 μM. The concentration of reagents in the tests with inhibitors was as follows: 10 μM bortezomib (tebu-bio), 50 μM leupeptin (Roth), and 10 μM activators. The release of the aminomethylcoumarin (AMC) reporter group was followed by monitoring the fluorescence emission at 460 nm (λex was 380 nm). DabEDS hydrolysis was detected by measuring the emission at 493 nm, while the excitation wavelength was set at 335 nm. Fluorescence measurements were carried out using an Infinite M200 Pro plate reader (Tecan), every 2 min for up to 60 min at 37 °C. All activity assays were performed in at least three independent replicates. The activity was calculated in relation to the catalytic activity of the vehicle (DMSO)-treated latent proteasome, which was regarded as 100%. Statistical analysis was performed using the one-way ANOVA followed by Tukey’s post hoc test. *p* value <0.05 was considered statistically significant.

#### Degradation of Native and Oxidized Proteins by 20S Proteasome

Stock solutions of native human enolase 2 (Novus Biologicals) and α-synuclein (rPeptide) were diluted with ddH_2_O to 0.5 mg mL^–1^ and incubated with 0.5% H_2_O_2_ at 30 °C for 2 h. The reactions were stopped by the addition of DTT (12.5 mM), and then extensively dialyzed overnight against either a 10 mM Tris buffer pH 7.4 containing 50 mM KCl and 2.5 mM MgSO_4_ (enolase 2) or 20 mM Tris buffer pH 7.4 supplemented with 100 mM NaCl (α-synuclein). Human enolase 2 and its oxidized form (40 pmol) were dissolved in 20 mM Tris at pH 7.4. Native and oxidized α-synuclein (387 and 194 pmol, respectively) and native SOD1 (117 pmol, ProSpec-Tany TechnoGene Ltd.) and Tau-441 (61 pmol, rPeptide) were dissolved in 20 mM HEPES at pH 7.4. All samples were prepared by mixing h20S in a buffer consisting of 50 mM Tris/HCl pH 7.2, 0.1 mM EDTA, 1 mM DTT, 1 mM NaN_3_, 50% glycerol and 0.01% SDS, the protein solution in the assay buffer, and either DMSO (control) or the modulator dissolved in DMSO, to the total sample volume of 10 μL. The final concentration of DMSO did not exceed 0.05%. The samples were incubated at 37 °C for the time allowing us to observe changes in the rate of protein digestion by the proteasome alone and in the presence of modulators: 1.5 h for α-synuclein; 2 h for Tau-441, 3 h for enolase 2 and its oxidized form, 3 h for the oxidized form of α-synuclein, and 21 h for SOD1. The reaction was stopped with 4× Laemmli buffer and then heated at 75 °C for 10 min. The results were analyzed electrophoretically, after loading 8 μL of each sample onto 10% (enolase 2, oxy-enolase 2, and Tau-441) or 12% SDS–PAGE gels (α-synuclein, oxy-α-synuclein, and SOD1). The protein bands were detected with Coomassie Blue-based reagent, InstantBlue. The quantitative image analysis was carried out with the Quantity One 1-D analysis software (Bio-Rad) after the background intensity reduction. The amount of intact protein was calculated in relation to the protein incubated with h20S, considered to be 100%. Each value represents the average of three independent experiments. All results are presented as the mean ± SD. Statistical analysis was performed with Origin, using one-way analysis of variance (ANOVA) followed by the Bonferroni post hoc test for pairwise comparison. *p* value <0.05 was considered statistically significant.

#### Proteasome Activity in Cell Lysate

Human embryonic kidney cells (HEK293T) were grown in a T-75 flask to ∼80% confluency and maintained in Dulbecco’s modified Eagle medium (DMEM) supplemented with 10% fetal bovine serum and penicillin/streptomycin (100 units/mL/100 μg/mL), at 37 °C with 5% CO_2_. Then, the cells were lysed using Reporter Lysis Buffer (Promega, USA), according to the manufacturer’s protocol. The protein content was measured using the BCA assay. Stock solutions of the fluorogenic substrates (Suc-LLVY-AMC and Boc-LRR-AMC) and the tested compounds were prepared in DMSO. The final DMSO concentration was kept constant at 2%. The activators were used in five concentrations 1, 2.5, 5, 10, and 25 μM. The activity assays were performed in a 96-well plate format in 50 mM Tris–HCl buffer, pH 8.0, with a reaction volume of 100 μL. The final concentrations of the fluorogenic substrates and cell lysate proteins were 100 μM and 20 μg/mL, respectively. As the negative control, 1 μM bortezomib (ChT-L activity) or 50 μM leupeptin solution (T-L activity) was used. The release of AMC was measured continuously in 2 min intervals for 60 min, at 37 °C, using a Tecan Infinite M200 Pro spectrofluorimeter (Tecan Trading AG, Switzerland). The excitation and emission wavelengths were set at 380 and 460 nm, respectively.

#### Western Blot Analysis of the Protein Content in HEK293T Cells

Cellular models of Alzheimer’s disease (AD) and amyotrophic lateral sclerosis (ALS) were developed through transfection of HEK293T cells with plasmids encoding fusion proteins Tau-GFP (pLJM1 EGFP-Tau, Addgene no. 108868) or SOD1G37R-GFP (pF148 SOD1G37RAcGFP1, Addgene no. 26409). The TurboFect Transfection Reagent (ThermoFisher Scientific, no. R0533) was used according to the manufacturer’s instruction. At the time of transfection, cells were treated with DMSO (final concentration of 0.1%; control cells), indicated concentrations of the activators, or 10 μM MG-132 proteasome inhibitor. The treatment was conducted for 6 h, followed by medium exchange. The cells were then cultured for 18 h in complete media made of DMEM (high-glucose variant, Gibco-Invitrogen, Carlsbad, CA) supplemented with 10% heat-inactivated fetal bovine serum and antibiotics (100 U/mL penicillin, 100 μg/mL streptomycin, and 0.25 μg/mL of amphotericin B; Gibco-Invitrogen). The incubators were maintained at 37 °C, 95% humidity and 5% CO_2_ saturation. For protein abundance analyses, 6 × 10^5^ HEK293T-derived cells were passaged on plates (10 cm in diameter) and allowed to attach overnight. The efficiency of transfection was estimated by determining fractions (percentage) of cells positive to GFP fluorescence in fluorescent microscopic investigations conducted after transfection, as described previously.^52^ The transfection efficiency was 48 ± 8%, calculated together for all plasmids (as the efficiency was similar in each case). Cells were harvested through scraping in PBS, then lysed in the buffer consisting of 1% Triton X-100, 0.5 mM EDTA, 150 mM NaCl, 50 mM Tris, pH 7.5, and a mixture of protease and phosphatase inhibitors (Roche Applied Science, Penzberg, Germany; no. 05892791001 and no. 11873580001). After clearing the lysate by centrifugation, the supernatant was used for automated Western blotting (WES system with 12–230 kDa Separation Module (no. SM-W003) and Anti-Mouse Detection Module (no. DM-002), ProteinSimple, San Jose, California, USA) according to the manufacturer’s instruction. Staining with anti-β-actin antibody (1:25,000) was used as an internal control to normalize the results. Primary anti-GFP antibody (Santa Cruz Biotechnology, no. sc-9996) was utilized to quantitate the amounts of Tau-GFP and SOD1-GFP proteins. Each experiment was repeated three times, using independent cell cultures. All data are presented as the mean ± SD. Statistical analysis was performed using the one-way ANOVA followed by Tukey’s post hoc test. *p* value <0.05 was considered statistically significant.

#### Crystallization

Human 20S proteasome was crystallized by mixing 0.5 μL of the protein solution (7.5 mg mL^–1^) and 0.5 μL of a crystallization buffer in Chryschem sitting drop vapor diffusion plates over a 500 μL reservoir of a crystallization buffer (0.1 M BisTris pH 6.5, 0.2 M MgCl_2_, 10% (w/v) PEG 3350). Crystallization plates containing 20S proteasome crystals initially grown at 18 °C were gradually cooled down to 4 °C over a period of 24 h. The plates were then transferred to the cold room and equilibrated there for at least 6 h. After equilibration, the wells were opened and 1.0 μL of the reservoir solution was added to the drop. Then, 2.0 μL of a stabilization solution (0.1 M BisTris pH 6.5, 0.2 M MgCl_2_, 20% (w/v) PEG 3350) was added to the crystallization drop and the reservoir solution was exchanged into 500 μL of a cryo buffer (0.1 M BisTris pH 6.5, 0.2 M MgCl_2_, 25% (w/v) PEG 3350, 20% (v/v) MPD). The resealed drop was equilibrated for at least 48 h against the exchanged reservoir. A 100 mM stock solution of a modulator was prepared in DMSO. Prior to soaking, the peptide was diluted to 5 mM in the cryo buffer. Crystals were soaked by the addition of the 5 mM peptide solution in a 1:1 volume ratio to the crystallization drop at 4 °C. Crystals were incubated for at least 48 h in the resealed wells before they were harvested. Initial phases for human 20S proteasomes were determined by molecular replacement using the human 20S structure (PDB ID: 5le5(10)). X-ray diffraction data were processed with the AutoProc toolbox.^53,54^ The model was built and optimized by several rounds of interactive manual model building in Coot^55^ and the refinement in Refmac5.^56^

#### Evaluation and Statistics

Origin software was used for statistical evaluation. Appropriate tests were used for statistical verification. This was one-way ANOVA with the Bonferroni or Tukey post hoc test, as indicated in figure legends. Statistical significance was assumed from an error value of *p* (*p < 0.05; **p < 0.01; ***p < 0.001).

## Supporting Information Available

Molecular dynamic simulations of Blm-pep in the binding pockets of the human 20S proteasome; determination of EC50 for the best stimulators; velocity of substrate degradation by h20S alone and in the presence of the best stimulators; comparison of stimulating capacity of the selected Blm-pep analogs, probed using substrates specific for chymotrypsin-like, trypsin-like, and caspase-like activity of h20S; sample traces of fluorescence intensity resulted from degradation of ChT-L substrate by human 20S proteasome alone and in the presence of the selected compounds; sample traces of fluorescence intensity resulted from the degradation of the FRET-type polypeptide substrate, Lys(Dabcyl)-Met-Ser-Gly-Phe-Ala-Ala-Thr-Ala-Glu(EDANS)-Gly, by human 20S proteasome alone and in the presence of the selected compounds; representative original SDS-PAGE gels presenting protein degradation by 20S proteasome stimulated by the modulators; representative full-size Western blots; velocity of Suc-LLVY-AMC substrate degradation by human 20S and 26S proteasomes in the presence of compounds **17** and **18**; Crystallographic data collection and refinement statistics (PDF)

## Accession Codes

PDB code for the structure of human 20S proteasome in the complex with **18**: 8BZL.

## Abbreviations

AD: Alzheimer’s disease
ALS: Amyotrophic lateral sclerosis
AMC: Aminomethylcoumarin
C-L: Caspase-like activity
ChT-L: Chymotrypsin-like activity
CP: Core particle
DabEDS: Lys(Dabcyl)-Met-Ser-Gly-Phe-Ala-Ala-Thr-Ala-Glu(EDANS)-Gly FRET-type proteasome substrate
FRET: Förster resonance energy transfer
GFP: Green fluorescence protein
HEK: Human embryonic kidney
HbYX: Moiety consisting of Hb-hydrophobic
Y: Tyrosine
and X: Any amino acid
h20S: Human 20S proteasome
PD: Parkinson’s disease
SD: Standard deviation
TFA: Trifluoroacetic acid
T-L: Trypsin-like activity
