# Supplementary material for: Blm10-Based Compounds Add to the Knowledge of How Allosteric Modulators Influence Human 20S Proteasome
Source: ACS Chem Biol. 2025 Feb 5;20(2):266–80. doi: 10.1021/acschembio.4c00341 (PMC11851449; doi:10.1021/acschembio.4c00341)
Supplement: Supplementary file 1 — cb4c00341_si_001.pdf [file cb4c00341_si_001.pdf]

Supporting Information

Blm10-based compounds add to the knowledge of how allosteric modulators influence human 20S proteasome

Julia Witkowska[1#], Małgorzata Giżyńska[1#], Przemysław Karpowicz[1], Daria Sowik[1], Karolina Trepczyk[1], Fabian Hennenberg[2], Ashwin Chari[2,3], Artur Giełdoń[4], Karolina Pierzynowska[5], Lidia Gaffke[5], Grzegorz Węgrzyn[5], Elżbieta Jankowska*[1]

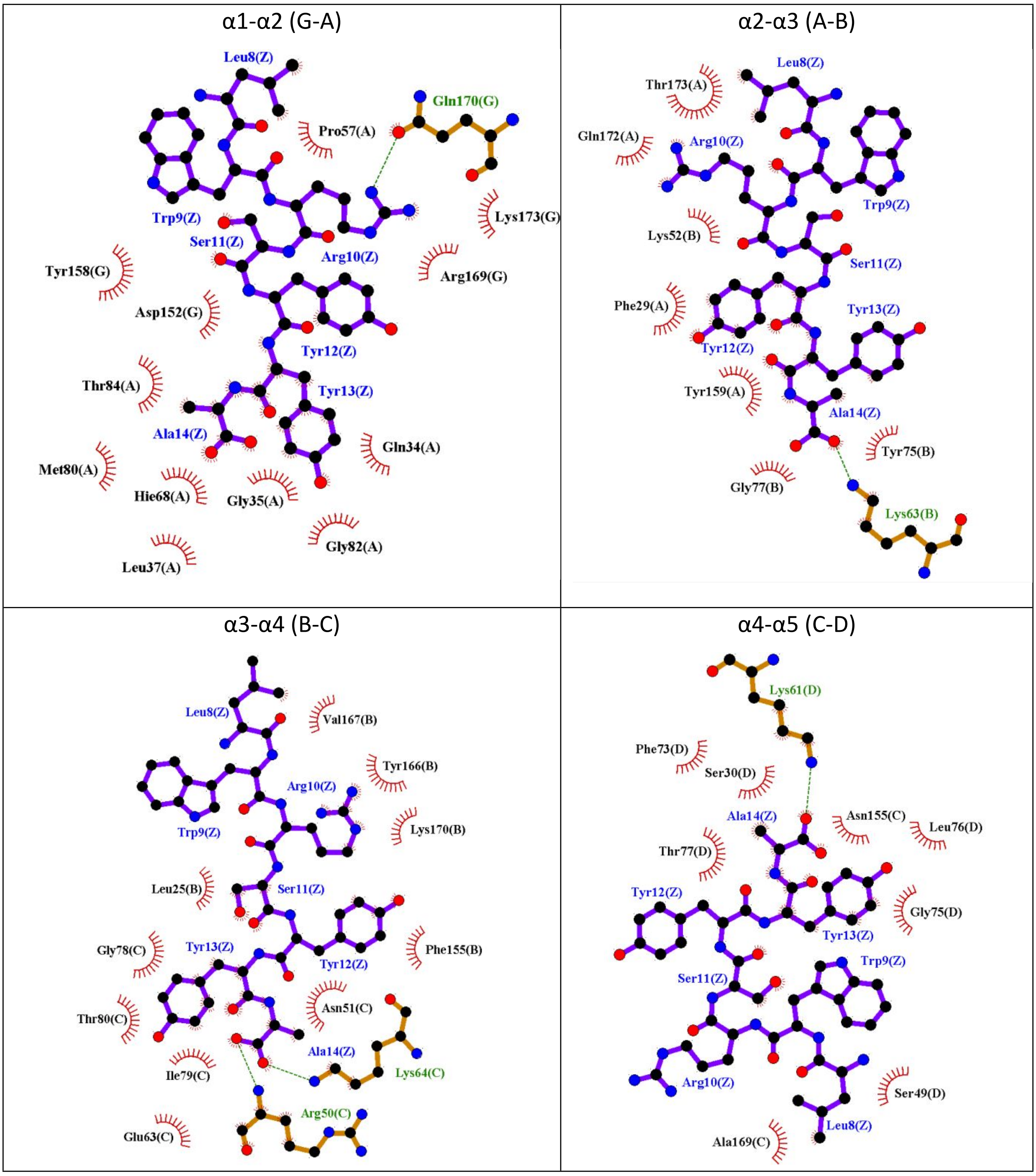
α1-α2 (G-A)
Leu8(Z)
Gln170(G)
Pro57(A)
Trp9(Z)
Ser11(Z)
Arg10(Z)
Lys173(G)
Tyr158(G)
Arg169(G)
Asp152(G)
Tyr12(Z)
Thr84(A)
Tyr13(Z)
Ala14(Z)
Gln34(A)
Met80(A)
Hie68(A)
Gly35(A)
Gly82(A)
Leu37(A)
α2-α3 (A-B)
Thr173(A)
Leu8(Z)
Gln172(A)
Arg10(Z)
Trp9(Z)
Lys52(B)
Ser11(Z)
Phe29(A)
Tyr13(Z)
Tyr12(Z)
Tyr159(A)
Ala14(Z)
Tyr75(B)
Gly77(B)
Lys63(B)
α3-α4 (B-C)
Leu8(Z)
Val167(B)
Tyr166(B)
Arg10(Z)
Trp9(Z)
Lys170(B)
Leu25(B)
Ser11(Z)
Gly78(C)
Tyr13(Z)
Tyr12(Z)
Phe155(B)
Thr80(C)
Asn51(C)
Ala14(Z)
Lys64(C)
Ile79(C)
Arg50(C)
Glu63(C)
α4-α5 (C-D)
Lys61(D)
Phe73(D)
Ser30(D)
Ala14(Z)
Asn155(C)
Leu76(D)
Thr77(D)
Tyr12(Z)
Gly75(D)
Tyr13(Z)
Trp9(Z)
Ser11(Z)
Arg10(Z)
Ser49(D)
Leu8(Z)
Ala169(C)

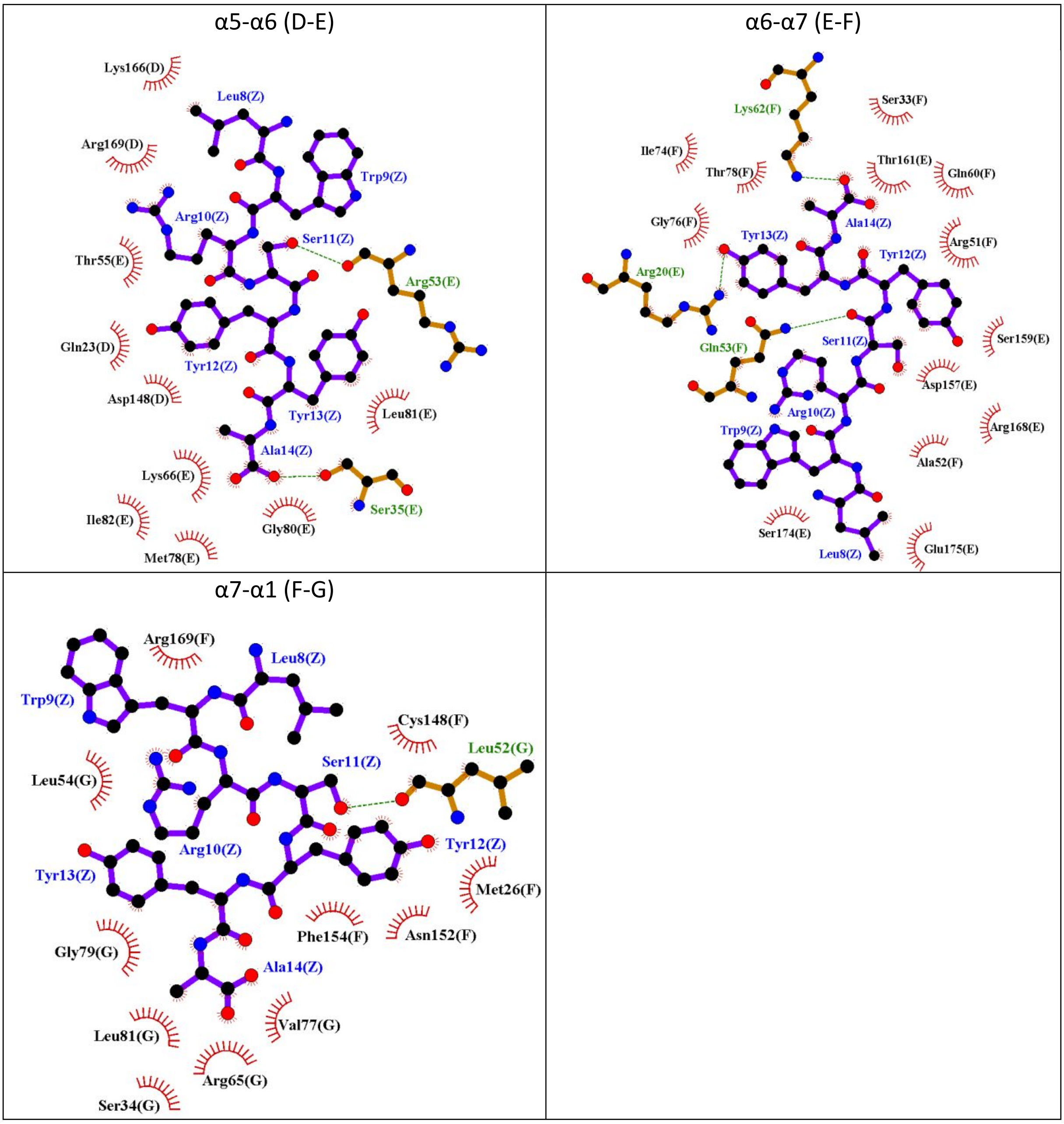

Fig. S1. The putative interactions of Blm-pep with the α pockets of human 20S proteasome (PDB ID: 4R3O). The initial position of the ligand was taken from PDB ID: 4ZZG (yeast 20S proteasome complexed with Blm-pep). The α subunits of the human proteasome (PDB ID: 4R3O) were superimposed to the yeast counterparts to get the initial structure for the modeling process. The modeling results were visualized by LigPlot program.

A

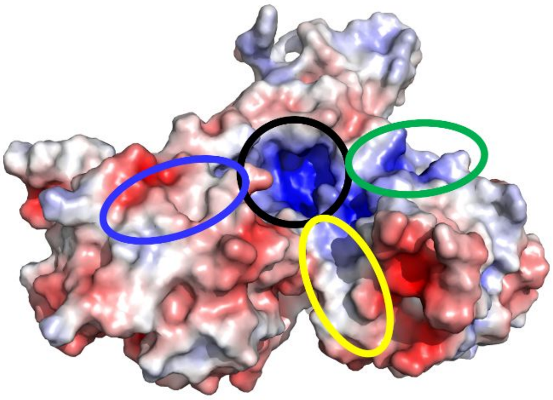

B

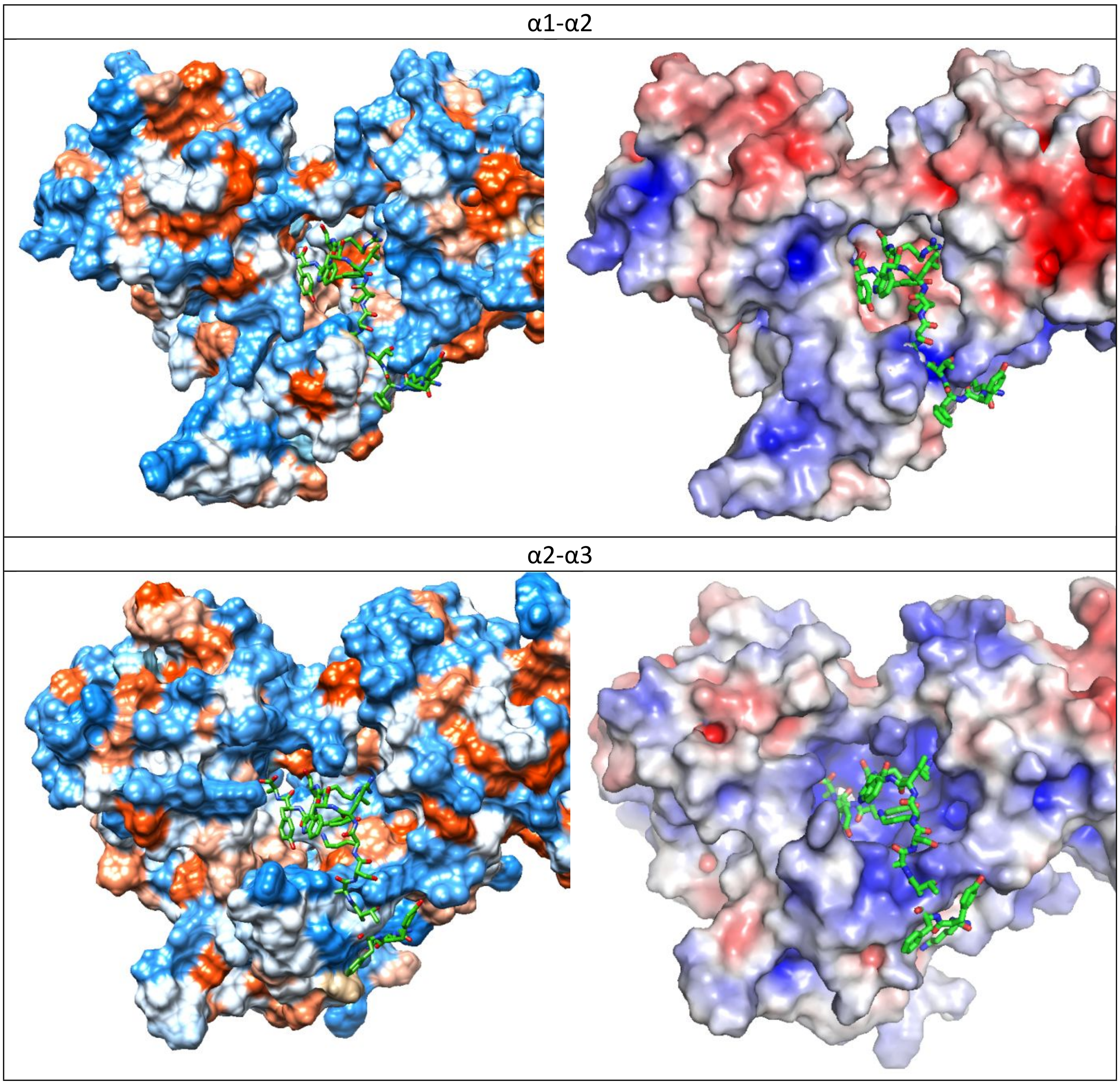

α3-α4

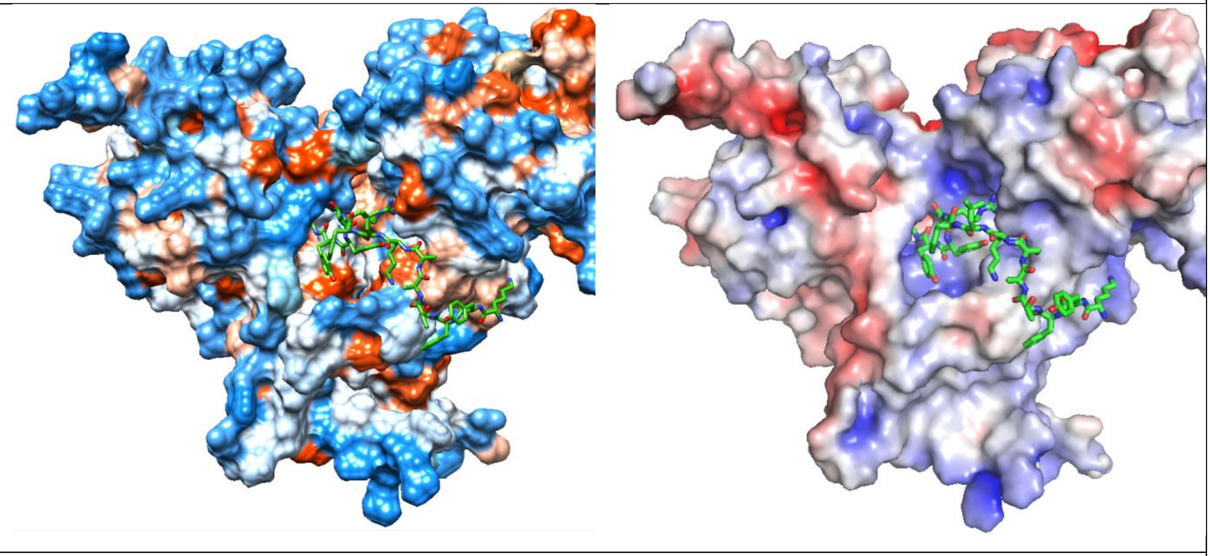

α4-α5

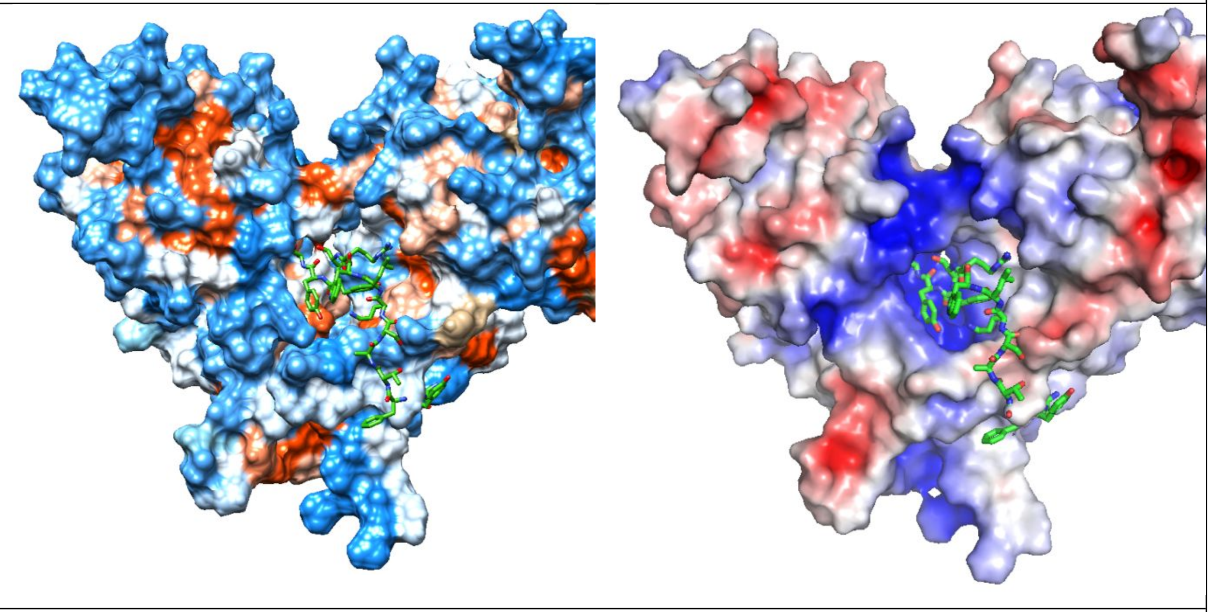

α5-α6

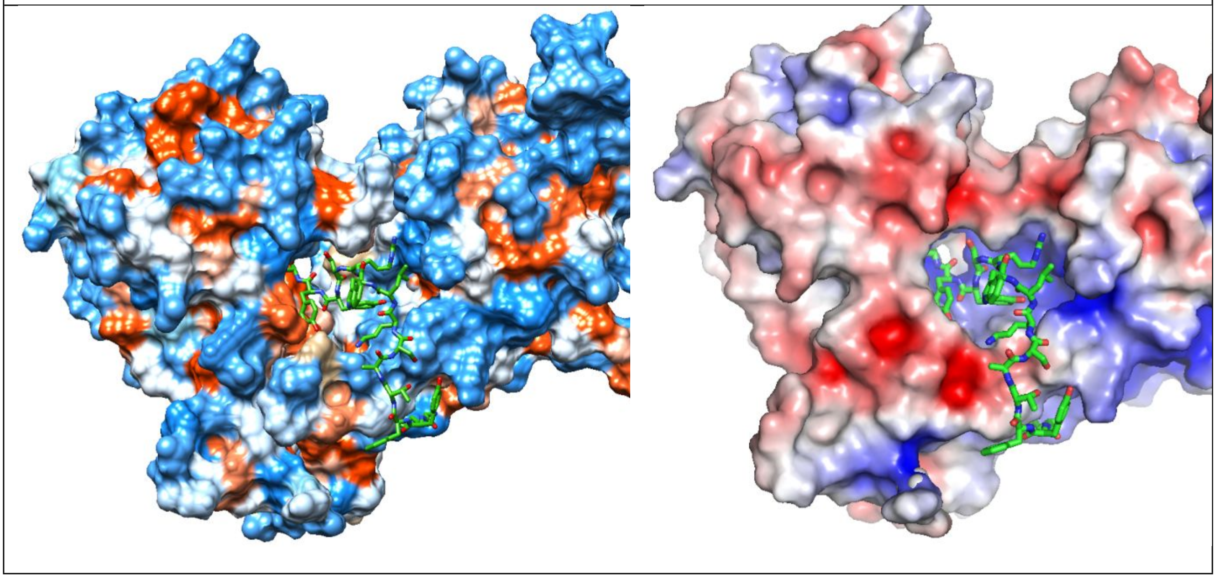

α6-α7

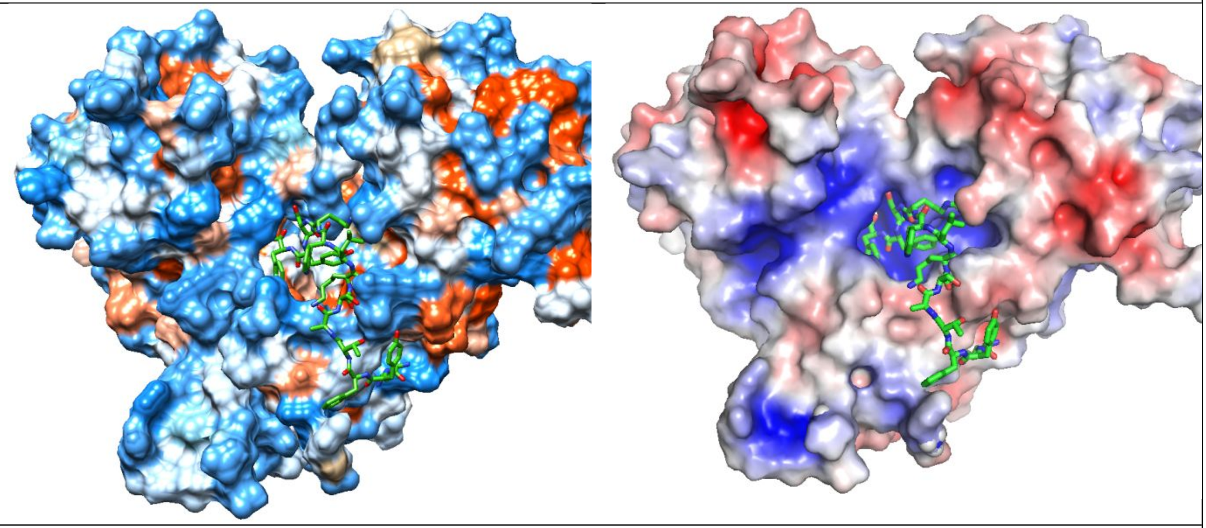

α7-α1

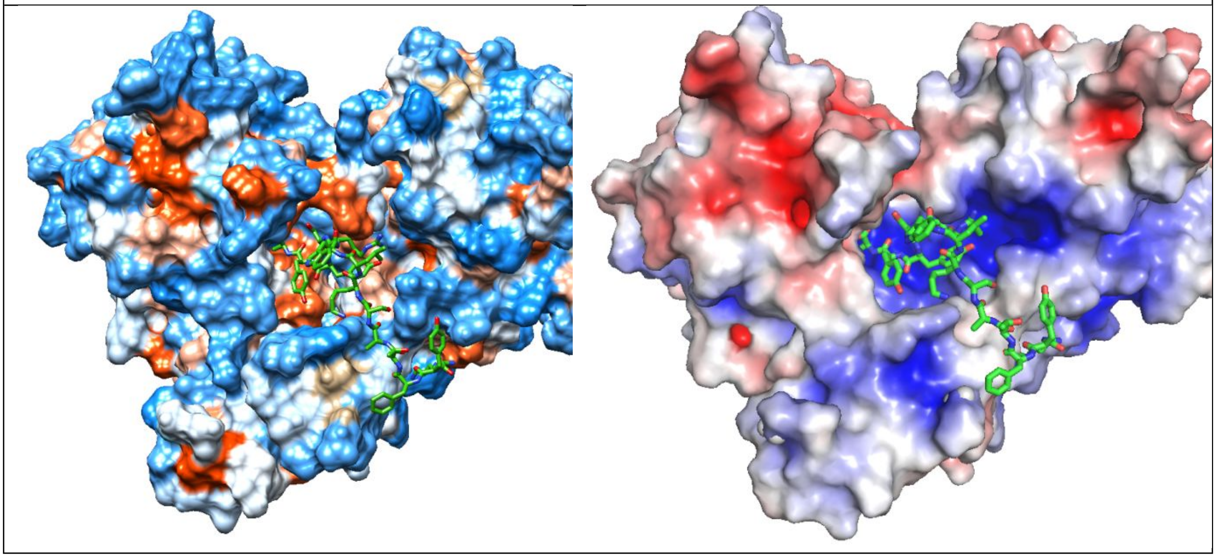

C

α1-α2

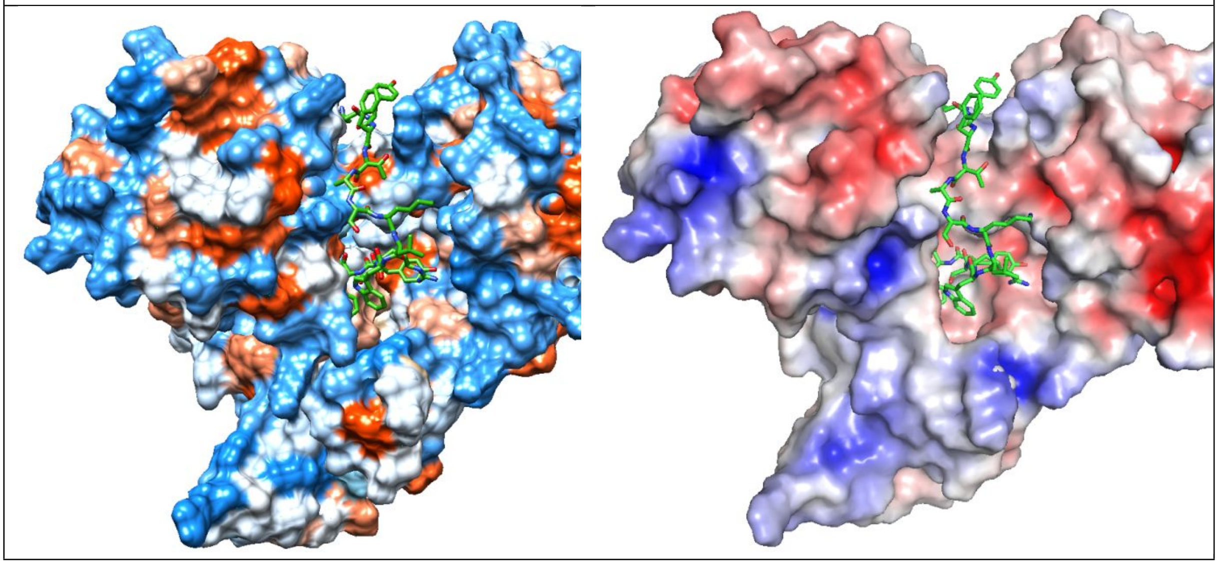

α2-α3

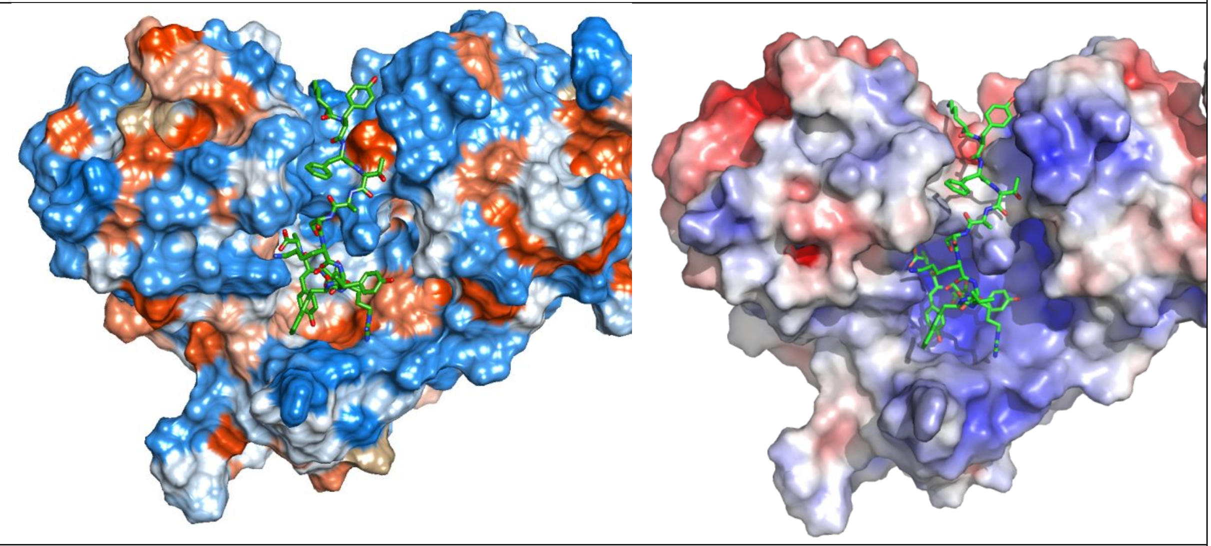

α3-α4

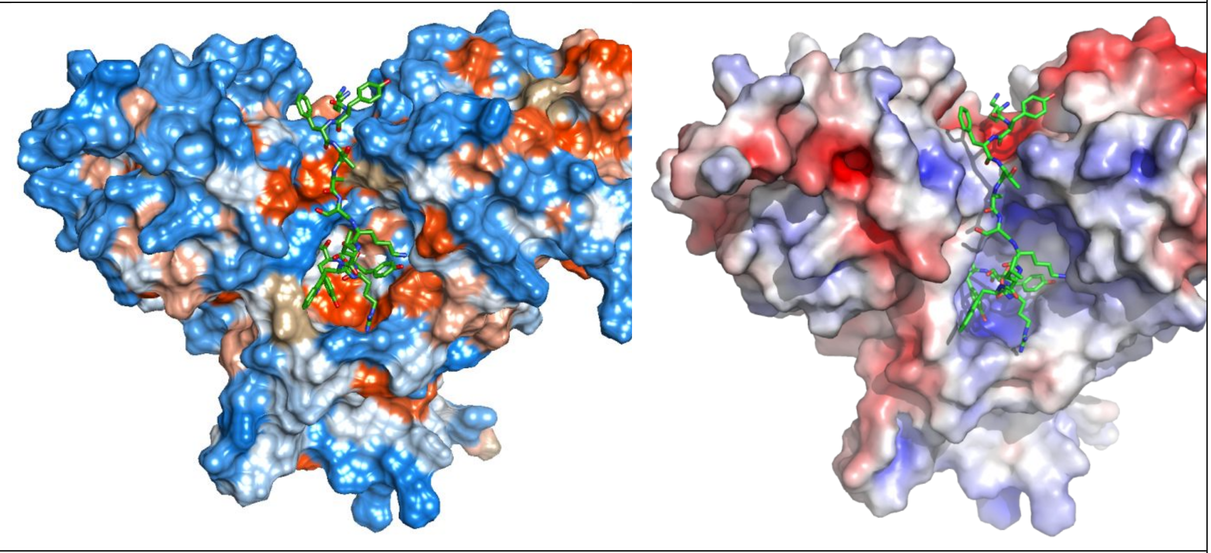

α4-α5

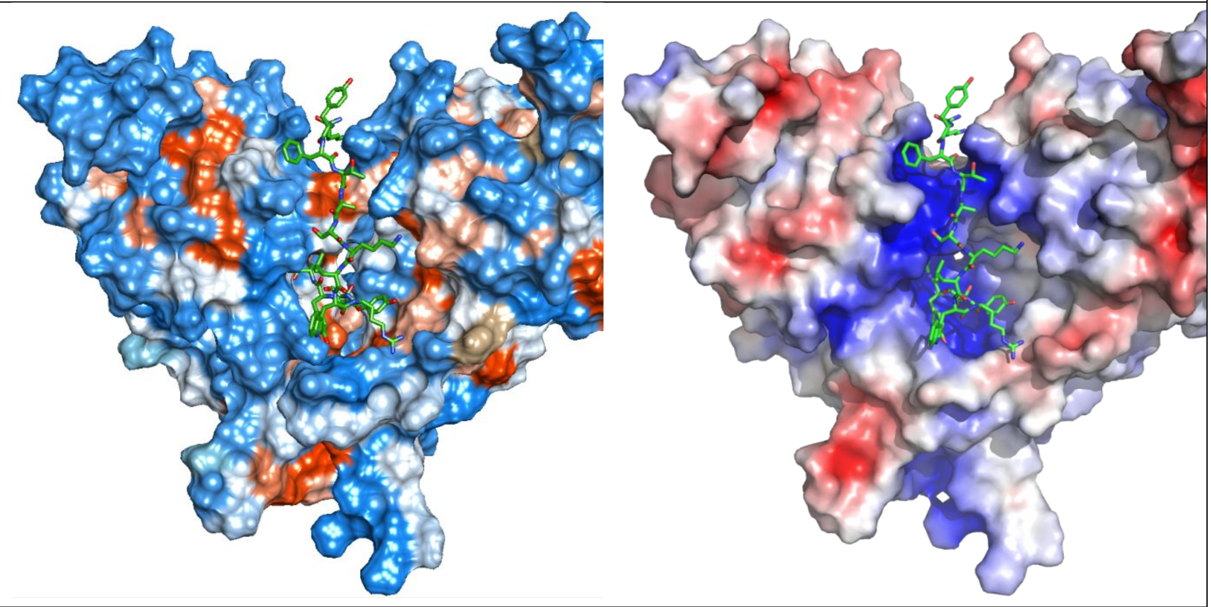

α5-α6

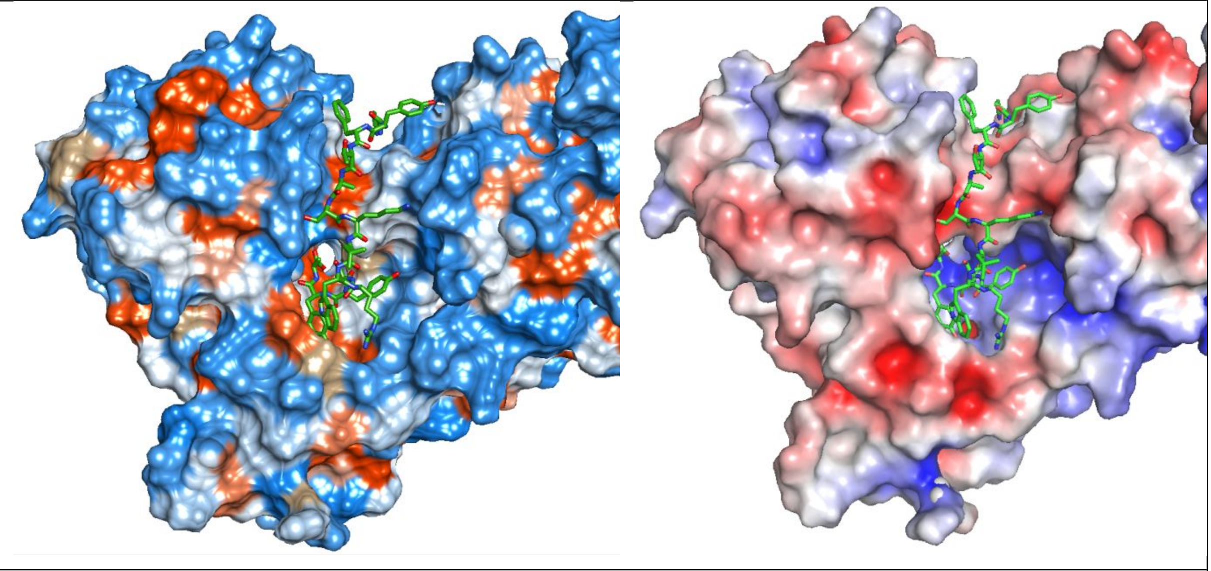

α6-α7

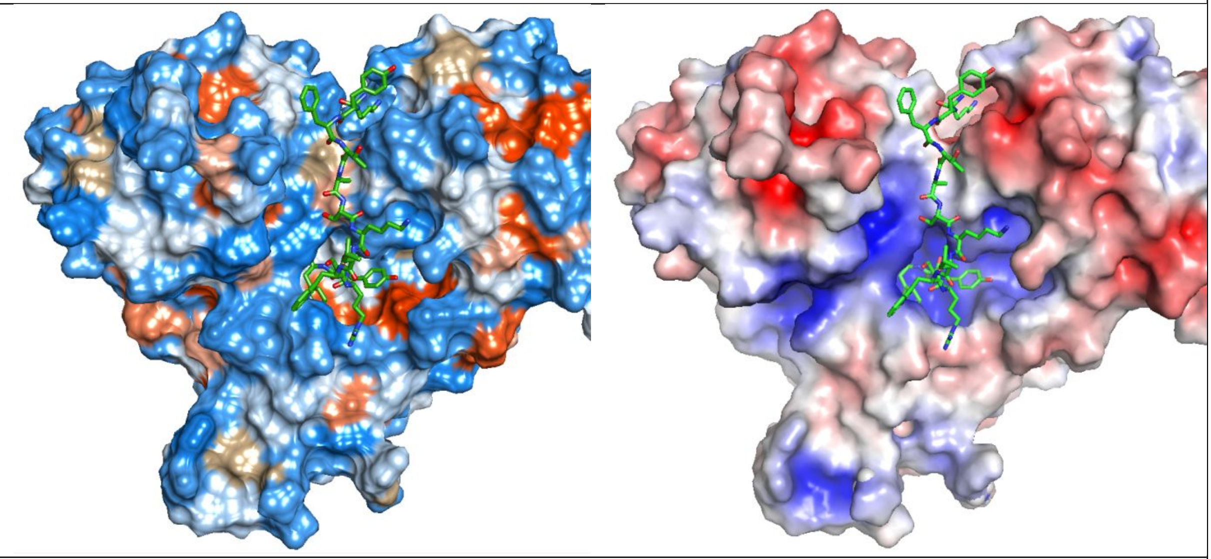

α7-α1

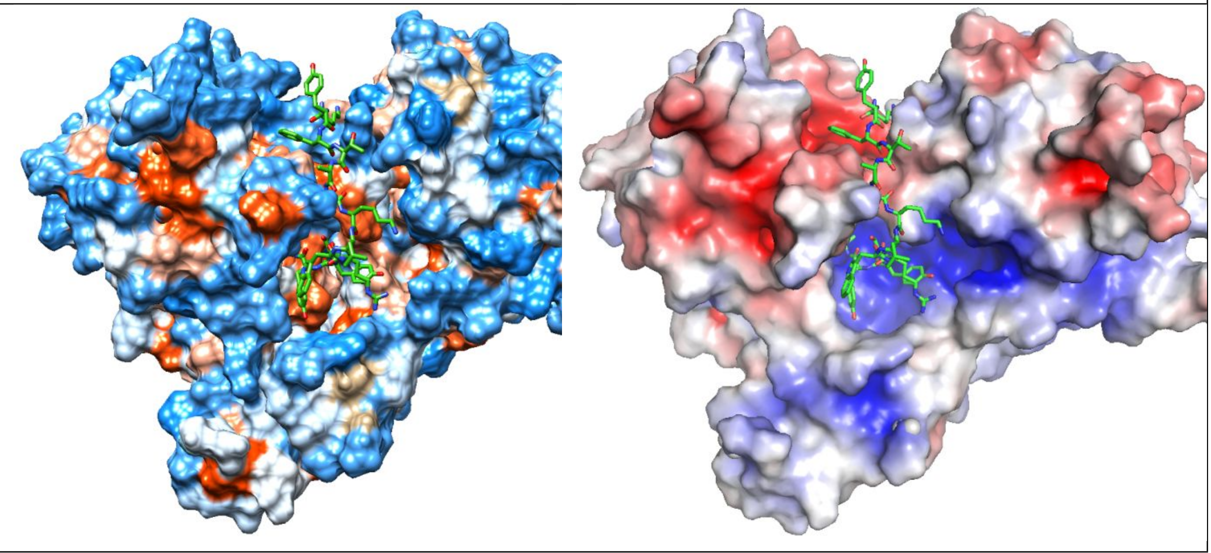

Fig. S2. **A**. Postulated directions in which the N-terminal region of Blm modulators may interact with the α ring of the human 20S proteasome. The black circle indicates the binding pocket located between the α subunits. The green, blue and yellow colors indicate the directions of the Blm chain, the α gate and the α rim, respectively. **B.** and **C**. Modeling Blm-pep interactions with the human 20S proteasome. The initial position of the ligand was taken from PDB ID: 4ZZG (yeast 20S proteasome complexed with Blm-pep). The α subunits of the human proteasome (PDB ID: 4R3O) were superimposed to the counterparts to get the initial structure for the modeling process. The modulator's N-terminal fragment missing in 4ZZG structure was constructed with PyMol software and docked on the surface of 4R3O **B.** towards the α gate, **C.** towards the α rim. One substitution in comparison to the original Blm-pep sequence was made: alanine was placed in position 5 instead of glycine to get information on possible contacts of this residue. The newly obtained complexes were optimized with the AMBER forcefield. Low temperature molecular dynamics and structure minimization in repetitive cycles were used. The obtained results were visualized by using UCSF Chimera and PyMol program. The electrostatic surface was calculated using APBS, the adaptive Poisson-Boltzmann solver [https://doi.org/10.1002/pro.3280] as implemented in the PyMol. Visualization of the electrostatic potential of the proteasome α subunits. The blue region shows the location of positive electrostatic potential, while the red region is the location of negative electrostatic potential. Calculations were performed at pH = 7.0. The hydrophobicity properties of amino acids are indicated using the Kyte and Doolittle hydrophobicity scale; the most polar residues are in medium purple, and the most hydrophobic residues are in blue in the surface representation.

Proteasome stimulating activity of the selected modulators

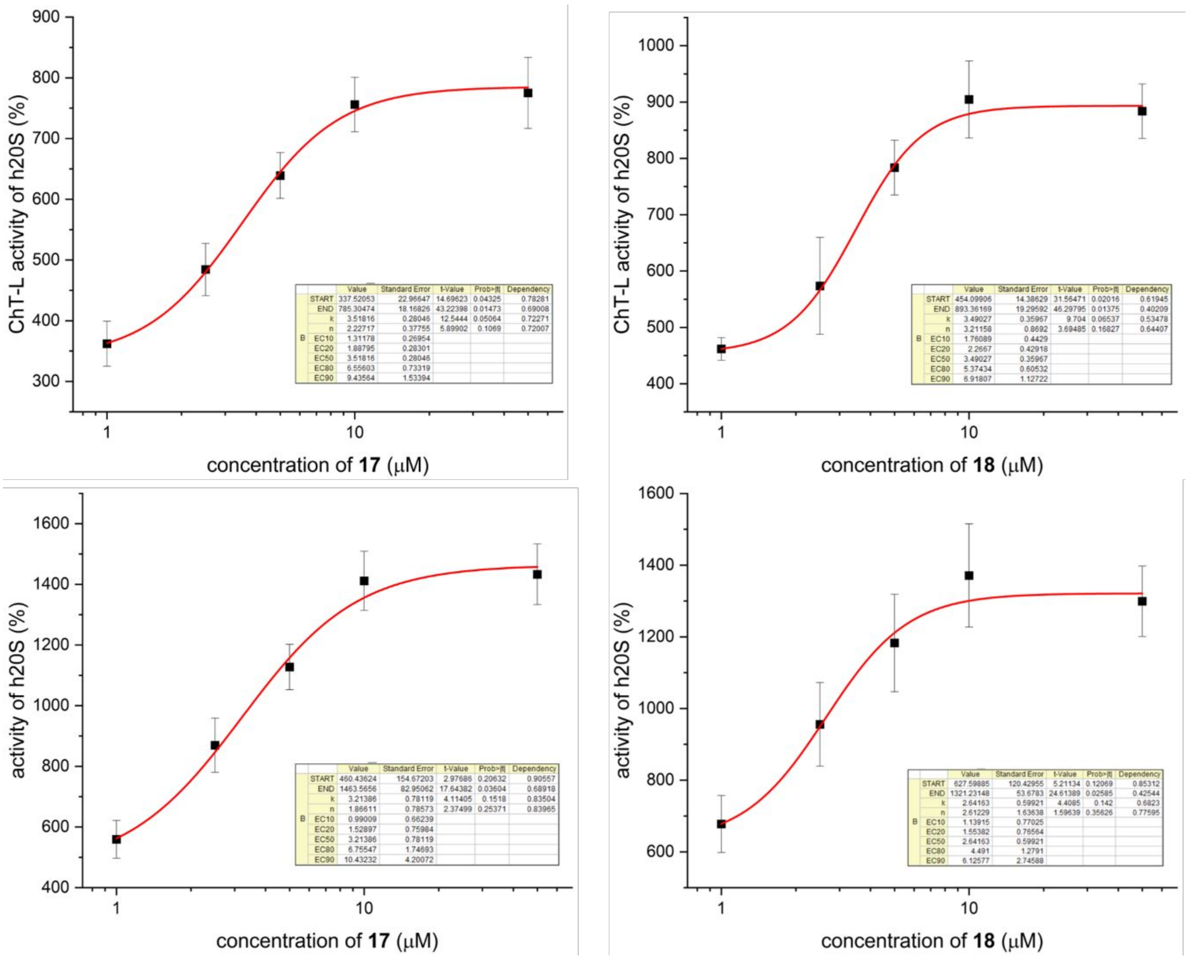

Fig. S3. Determination of EC50 (the compound concentration at which 50% of the maximal proteasome activation was achieved) for the best stimulators, compounds **17** and **18.** Titration curves present dependence of h20S activity, probed with either Suc-LLVY-AMC (upper panels) or Lys(Dabcyl)-Met-Ser-Gly-Phe-Ala-Ala-Thr-Ala-Glu(EDANS)-Gly (DabEDS; lower panels), on the concentration of compounds **17** and **18**. EC50 for **17** is 3.52 ± 0.28 μM when determined using Suc-LLVY-AMC and 3.21 ± 0.78 μM when determined using DabEDS substrate. EC50 for **18** is 3.49 ± 0.36 μM and 2.64 ± 0.60 μM, respectively. Results are the means of at least three independent experiments performed in two technical repeats (error bars represent standard error of the mean, SEM).

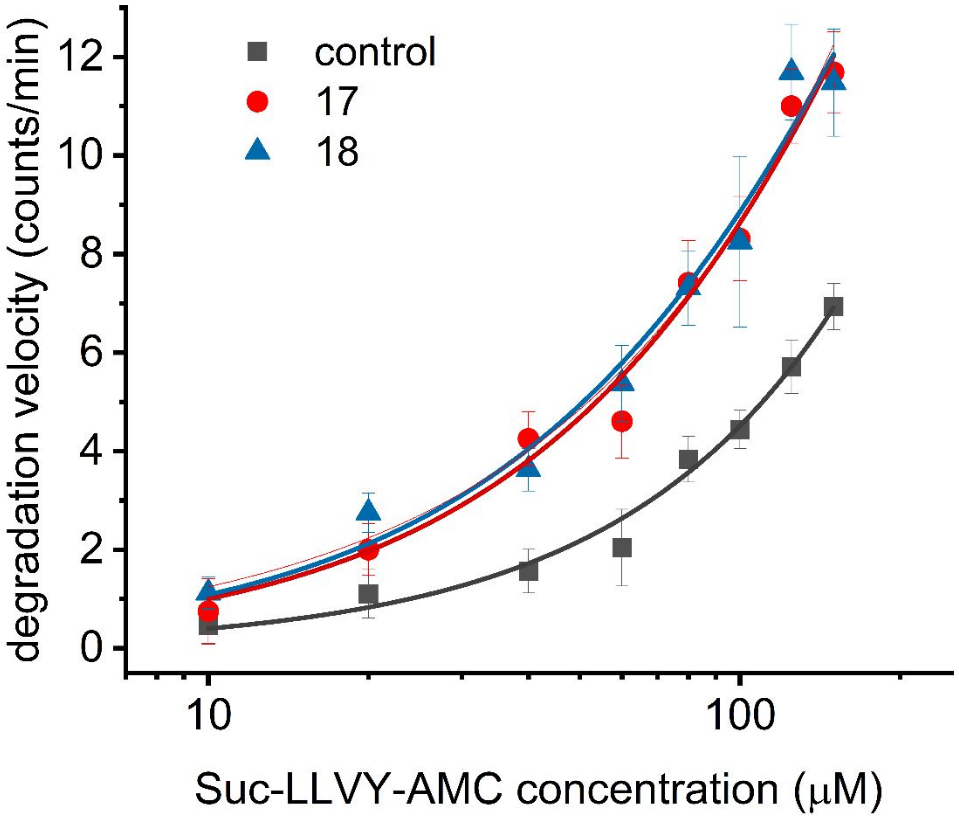

Fig. S4. Velocity of substrate degradation by h20S alone and in the presence of either **17** or **18** at a concentration of 10 μM. Results are the means of at least three independent experiments performed in two technical repeats (error bars represent standard error of the mean, SEM). Michaelis-Menten kinetics was applied for the fitting of the experimental data.

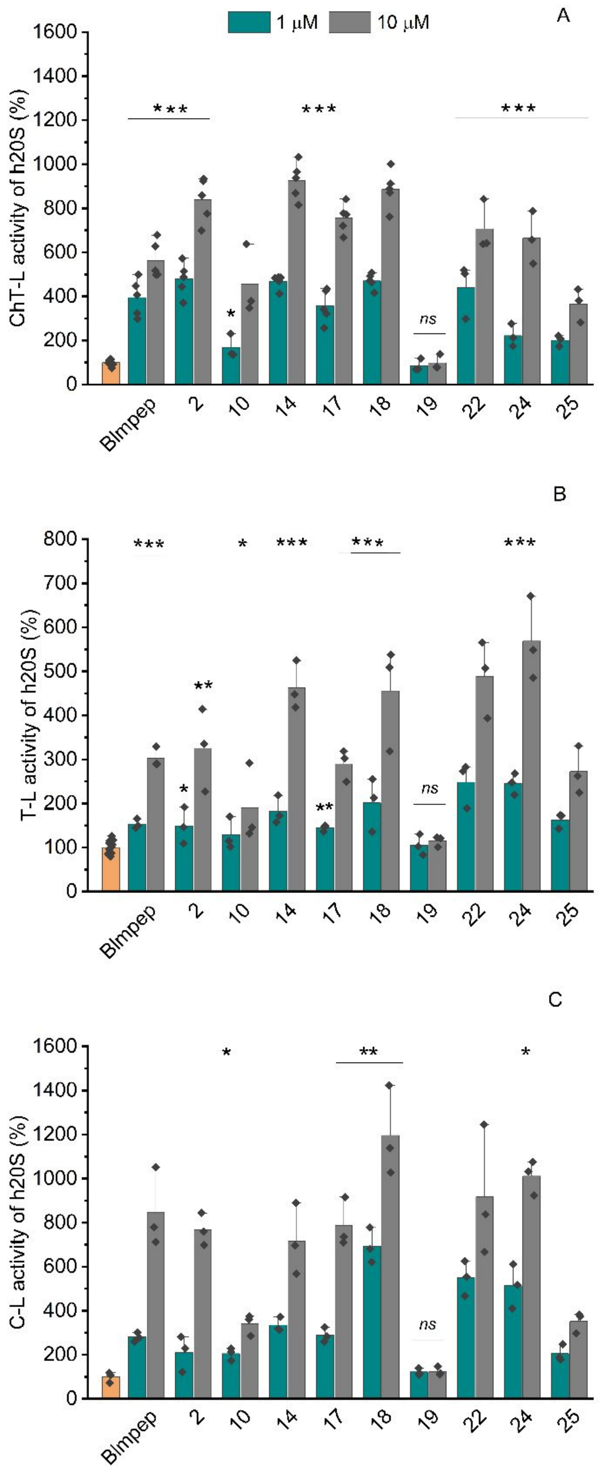

Fig. S5. Comparison of stimulating capacity of the selected Blm-pep analogs, probed using substrates specific for chymotrypsin-like (Suc-LLVY_AMC, 100 µM), trypsin-like (Boc-LRR-AMC, 100 µM) and caspase-like (Z-LLE-AMC, 100 µM) activity of human 20S proteasome. Results are the means of at least three independent experiments performed in two technical repeats (error bars represent standard error of the mean, SEM). One-way ANOVA with Tukey's post hoc tests was used to determine statistical significance of the observed differences (* $p<0.05$, ** $p<0.001$, *** $p<0.0001$).

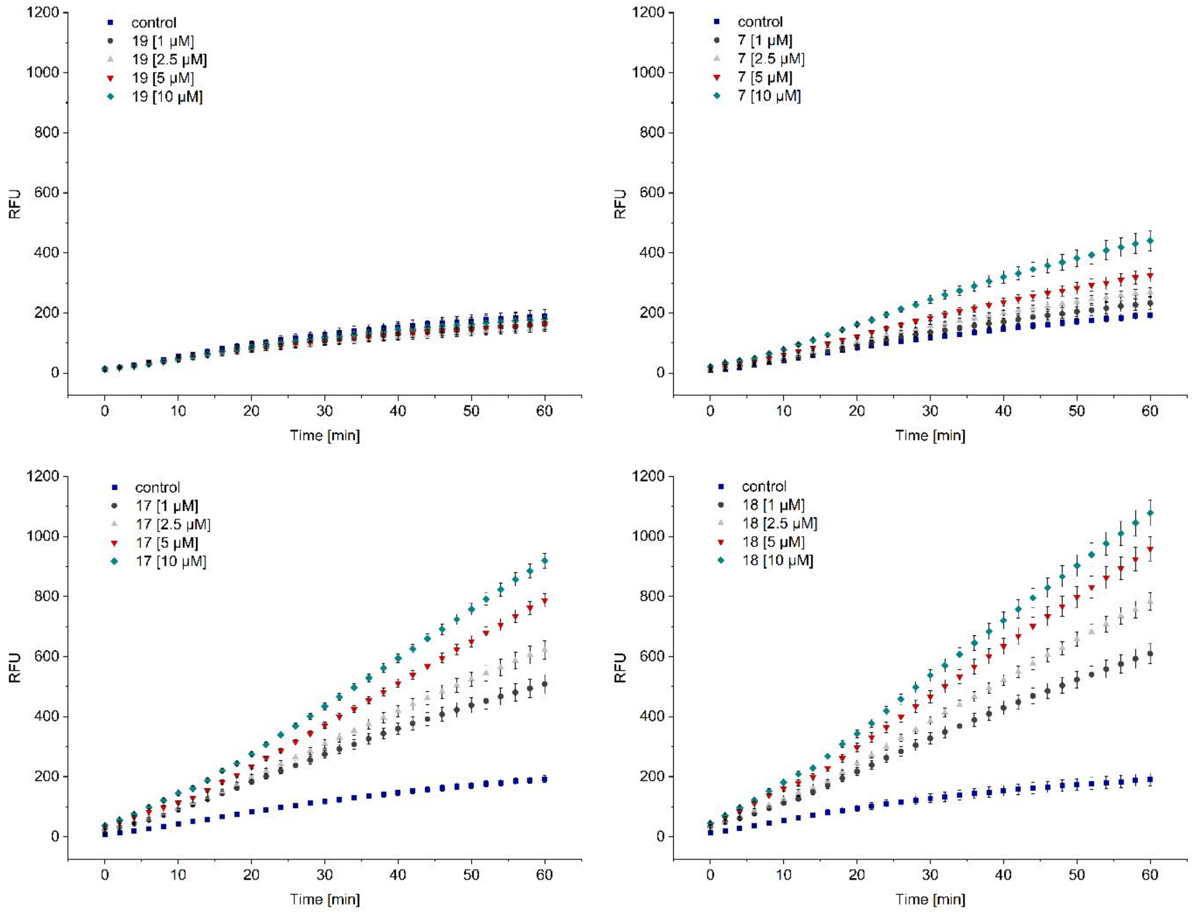

Fig. S6. Sample traces of fluorescence intensity resulted from degradation of Suc-LLVY-AMC by human 20S proteasome in the presence of the specified compounds: **19**, which is a negative control, and **7**, **17** or **18**, which are among the most effective stimulators of h20S activity. Results are the means of at least three independent experiments performed in two technical repeats (error bars represent standard error of the mean, SEM).

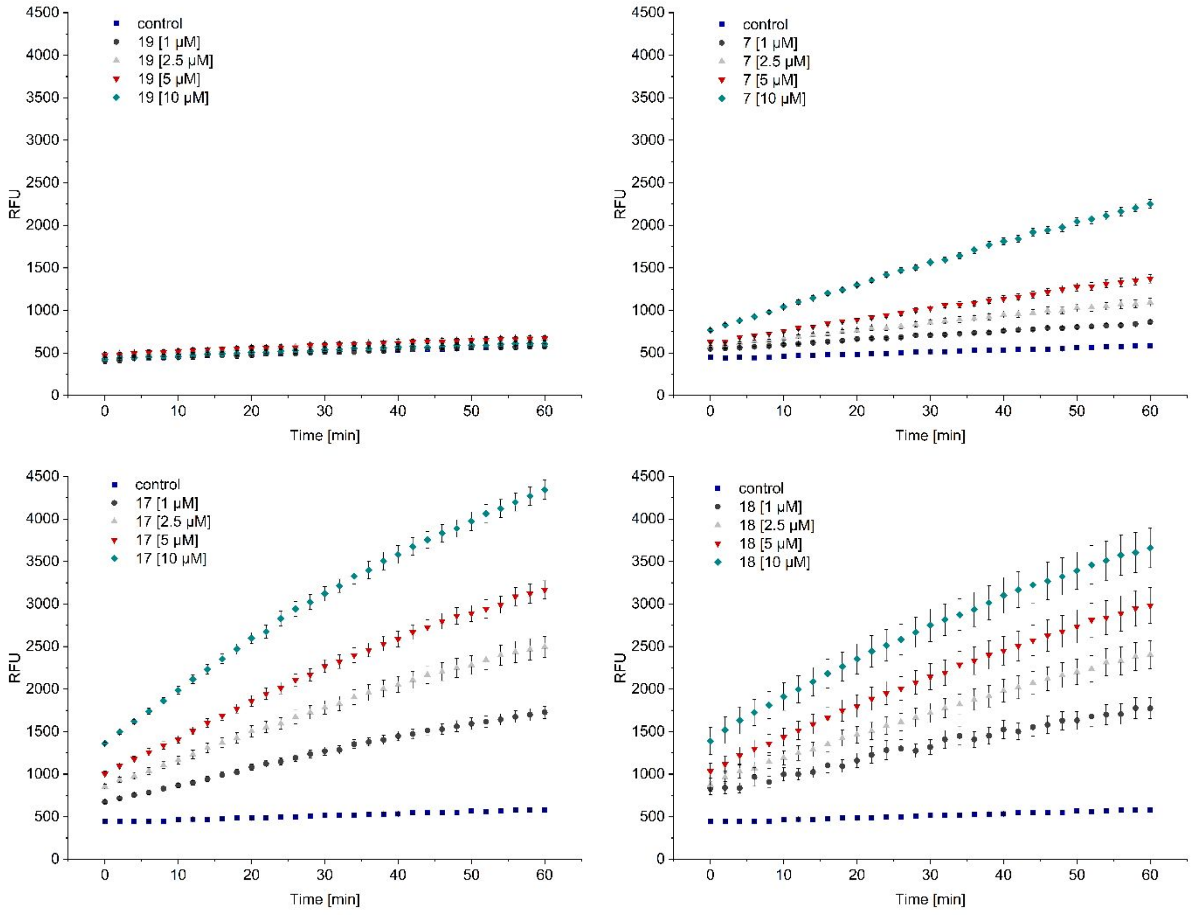

Fig. S7. Sample traces of fluorescence intensity resulted from degradation of polypeptidic FRET-type substrate, Lys(Dabcyl)-Met-Ser-Gly-Phe-Ala-Ala-Thr-Ala-Glu(EDANS)-Gly, by human 20S proteasome in the presence of the specified compounds: **19**, which is a negative control, and **7**, **17** or **18**, which are one of the most effective stimulators of h20S activity. Results are the means of at least three independent experiments performed in two technical repeats (error bars represent standard error of the mean, SEM).

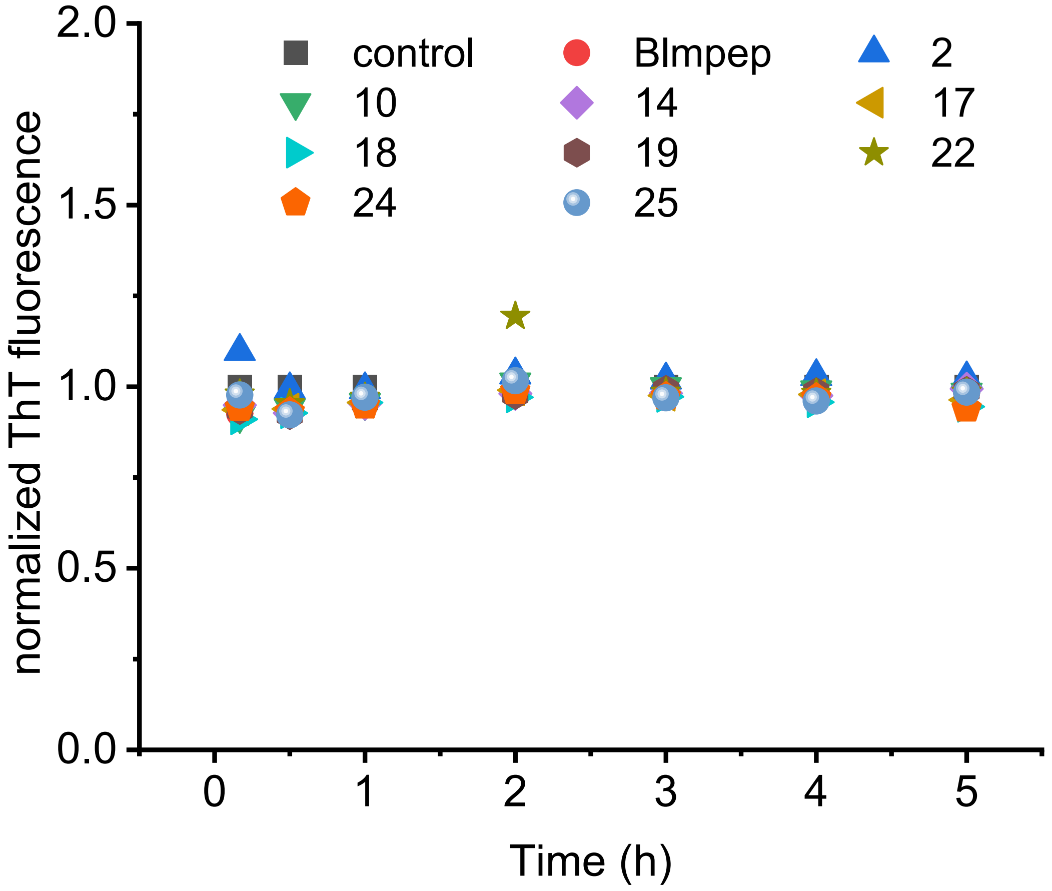

Figure S8. Lack of aggregation tendencies of modulators proven using thioflavin T assay. The assay was performed for the highest concentration of the peptides used in activity tests (10 μM). ThT concentration was 28 μM. Fluorescence intensity was measured after incubation of 10 μM modulators for the indicated time at 37°C. The buffer with 28 μM ThT was used as a control.

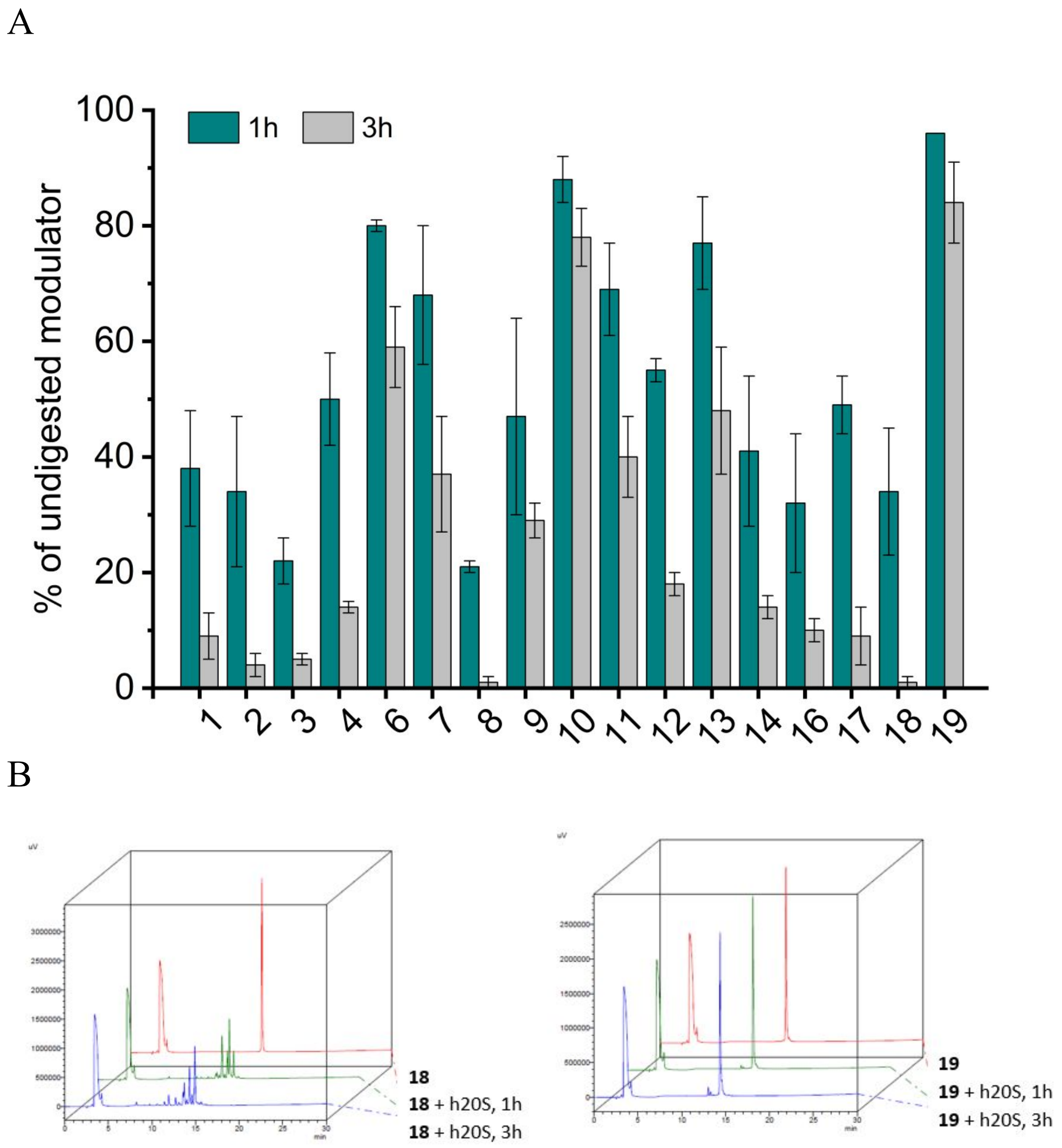

Figure S9. A. Susceptibility of modulators to be substrates of the human 20S proteasome, assessed using HPLC. Percentage of the undigested modulator was calculated based on the area under the peak corresponding to the modulator. B. Example chromatograms recorded for peptides alone (red) and peptides after incubation with h20S for 1 (green) or 3 hours (blue). The left panel shows strong degradation of compound **18**, which is an effective stimulator of the proteasome. The right panel shows that compound **19**, which has no ability to activate any of the h20S peptidases, is also not a substrate of the proteasome.

Protein degradation by h20S in the absence and presence of Blm modulators

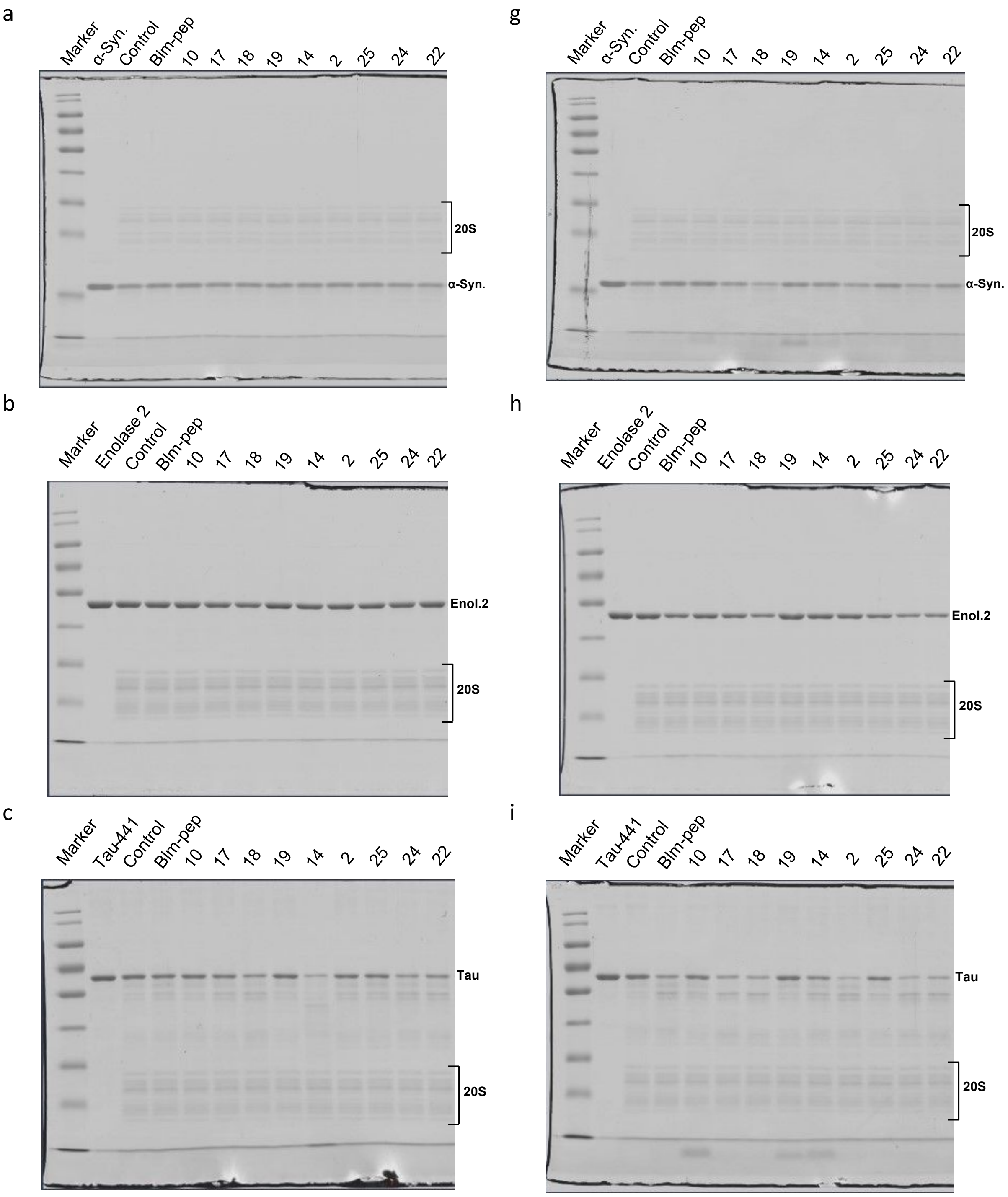

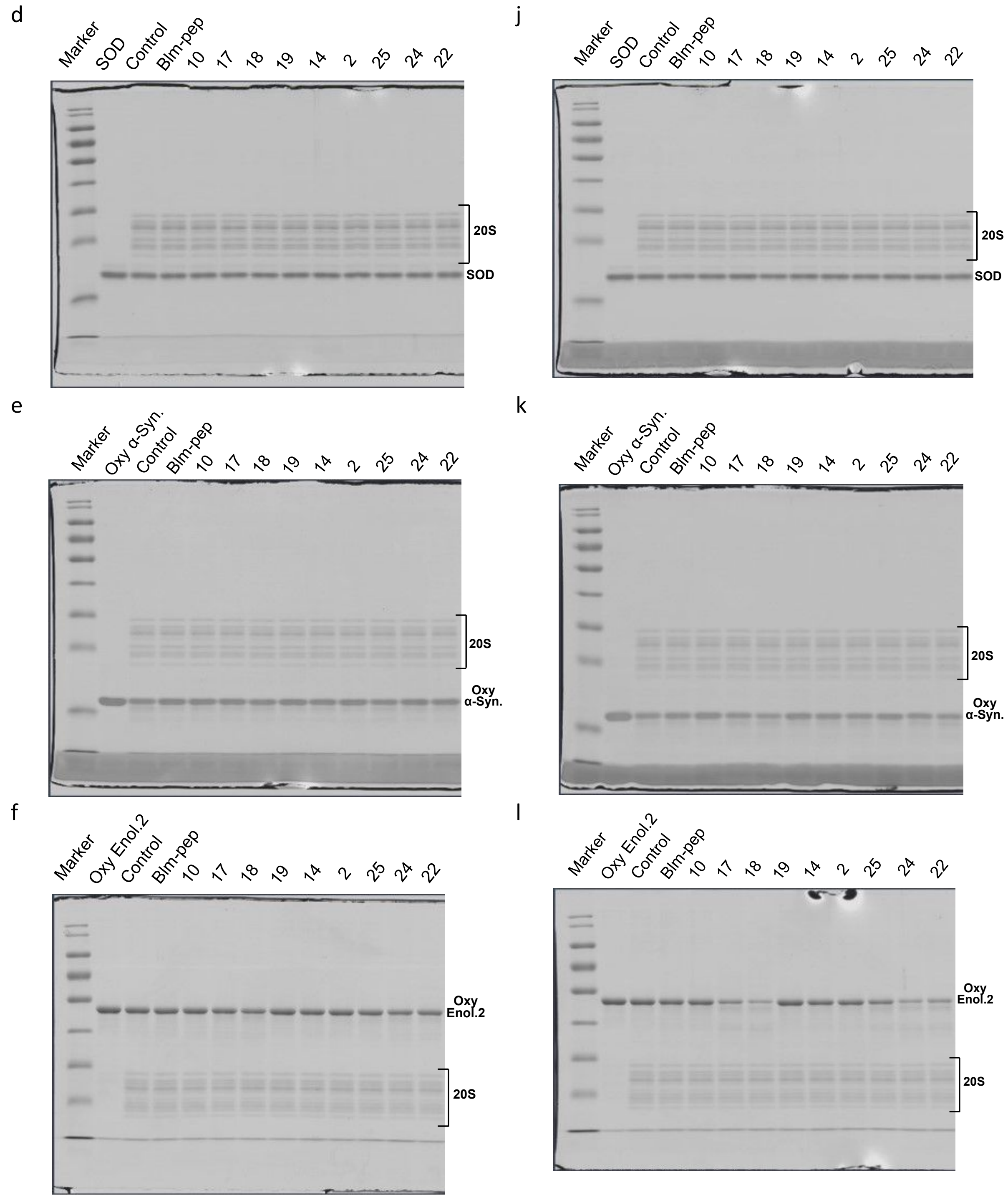

Figure S10. SDS PAGE gels of proteins degraded by h20S in the presence of either 1 (a-f) or 10 µM (g-l) concentration of Blm modulators. Proteasome subunits are indicated with brackets and serve as a loading control. PageRuler™ Prestained Protein Ladder (Thermo Scientific™) was used as a marker (bands: 180, 130, 100, 70, 55, 40, 35, 25, 15 and 10 kDa). Representative gels from three independent experiments are presented.

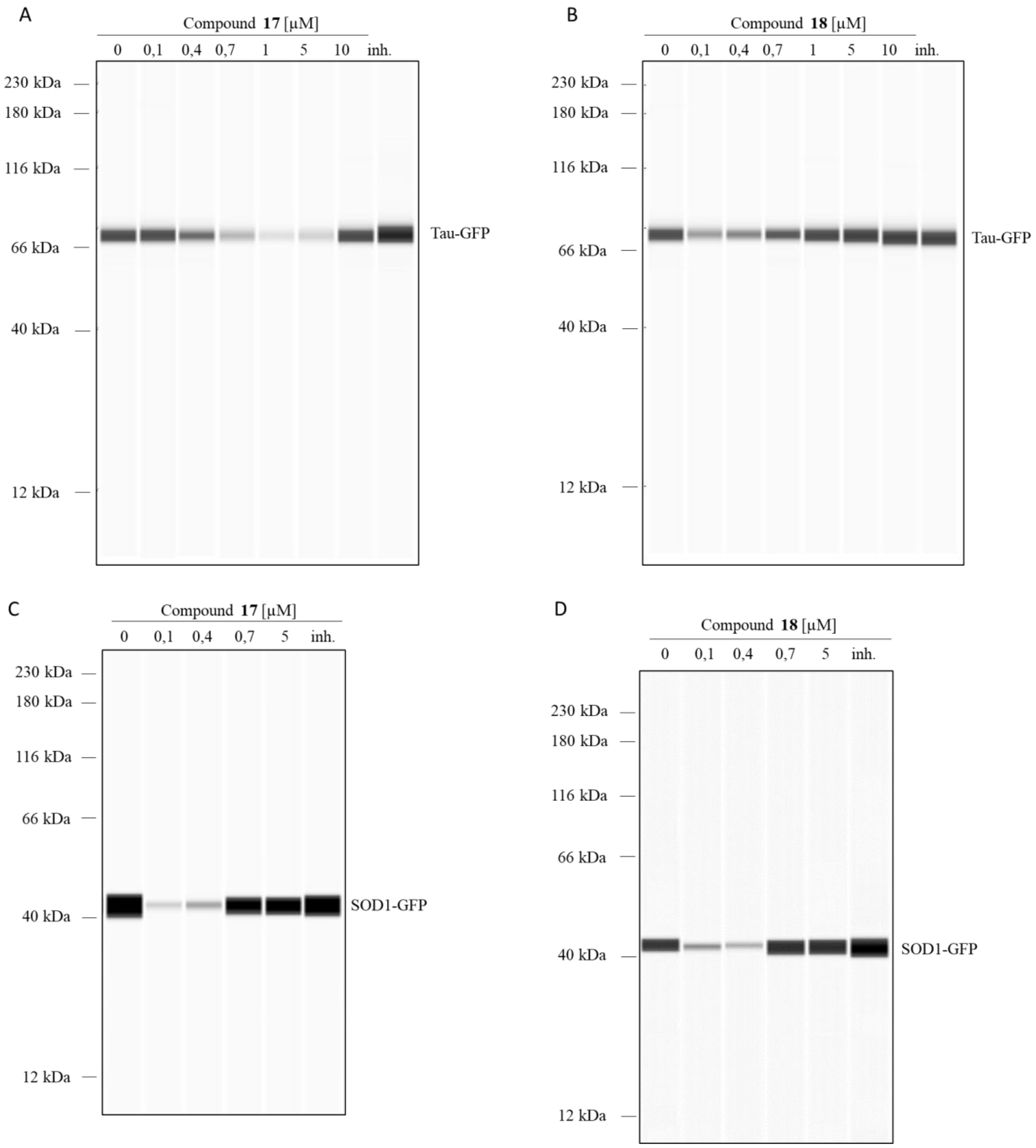

Figure S11. Representative full-size Western blots obtained using automatic capillary WES platform and specific anti-GFP antibodies. A. and B. The relative level of Tau-GFP in HEK293T cells after either **17** (A) or **18** (B) treatment for 24 h. C. and D. The relative level of GFP coupled to mutated SOD1 (SOD1G37R) in HEK293T cells after either **17** (C) or **18** (D) treatment for 24 h.

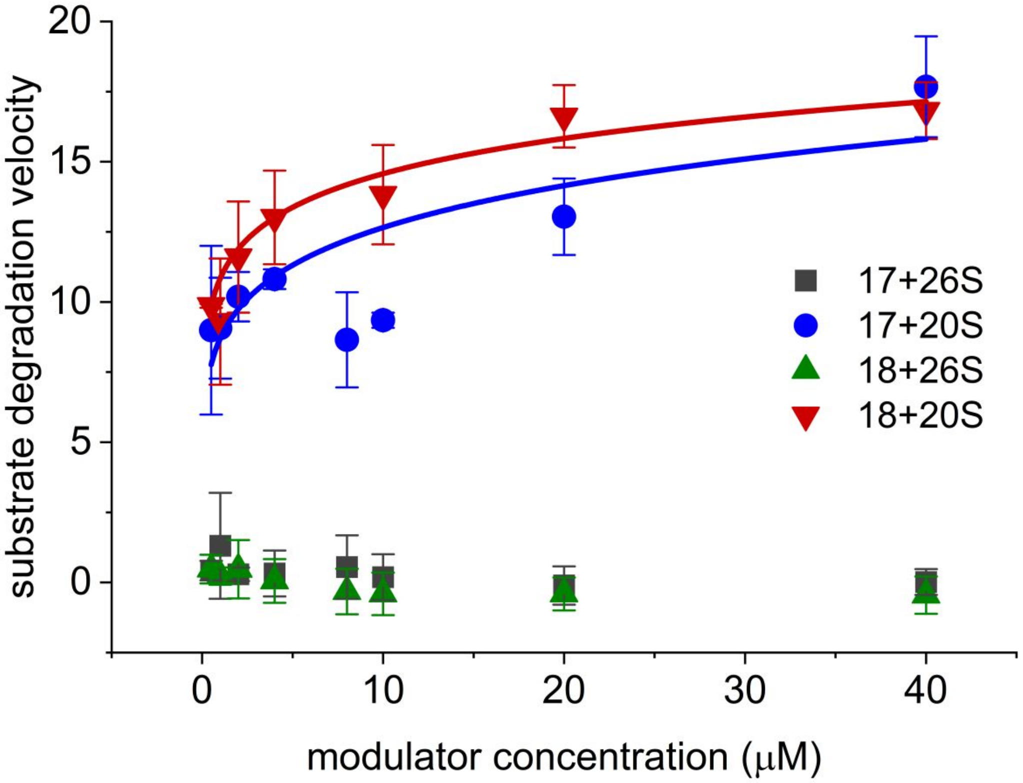

Fig.S12. The velocity of Suc-LLVY-AMC substrate degradation by human 20S and 26S proteasomes in the presence of compounds **17** and **18**. The assays were conducted in 96-well flat-bottomed black plates at 37°C. The assay buffer for h26S was 50 mM Tris pH 7.5 containing 40 mM KCl, 5 mM $MgCl_2$, 0.5 mM ATP, 1 mM DTT, 0.05 mg/ml BSA (ATP and DTT were freshly added). The assay buffer for h20S was 50 mM Tris pH 8.0. The proteasome concentration in the wells was 0.002 mg/ml. The final Suc-LLVY-AMC substrate concentration was 100 μM, and the modulators **17** and **18** were tested in the concentration range of 0.5 - 40 μM. Fluorescence measurements were made every 2 min for 1 h using a Tecan Infinite M200Pro plate reader (Tecan, Männedorf, Switzerland).

Table S1. Crystallographic data collection and refinement statistics for the complex of human 20S proteasome with compound **18.**

PDB ID	8BZL
Space group	P212121
Unit cell	
A (Å)	113.9
B (Å)	203.3
C (Å)	316.4
Data collection wavelength	0.976
Resolution range (highest resolution shell)	107.18 – 2.14 (2.30 – 2.14)
No. Of reflections	2327154
No. Of unique reflections	327921
Completness (spherical)	81.6 (21.7)
Completness (ellipsoidal)	94.5 (62.9)
<I/sI>	6.7 (1.5)
CC(1/2)	0.995 (0.636)
Rpim (all I+ & I-)	0.055 (0.558)
Rwork	0.226
Rfree	0.196
No. of. atoms	
Protein	47005
Ligand/solvent	3739
Activator peptide	205
Average B values (Å2)	
Protein	48
Activator peptide	69
Metals	48
Cl ions	66
Water	46
Wilson	48
R.m.s.d. bond length (Å)	0.0028
R.m.s.d bond angles (Å)	0.8923
Ramachandran % favoured	95.81
Ramachandran % outliers	0.63

* - values in parentheses are for the highest resolution shell
